# Clinical pharmacy key performance indicators for hospital inpatient setting: a systematic review

**DOI:** 10.1007/s11096-024-01717-x

**Published:** 2024-04-03

**Authors:** Lucas Magedanz, Hiolanda Lêdo Silva, Dayani Galato, Fernando Fernandez-Llimos

**Affiliations:** 1 Postgraduate Program in Health Sciences and Technologies, University of Brasília, Brasília, DF Brazil; 2 UCIBIO–Applied Molecular Biosciences Unit, i4HB–Institute for Health and Bioeconomy, Laboratory of Pharmacology, Faculty of Pharmacy, University of Porto, Porto, Portugal

**Keywords:** Health care quality indicators, Health care quality assurance, Health care outcome and process assessment, Hospital pharmacy service, Systematic reviews as topic

## Abstract

**Background:**

Key performance indicators (KPIs) are quantifiable measures used to monitor the quality of health services. Implementation guidelines for clinical pharmacy services (CPS) do not specify KPIs.

**Aim:**

To assess the quality of the studies that have developed KPIs for CPS in inpatient hospital settings.

**Method:**

A systematic review was conducted by searching in Web of Science, Scopus, and PubMed, supplemented with citation analyses and grey literature searches, to retrieve studies addressing the development of KPIs in CPS for hospital inpatients. Exclusions comprised drug- or disease-specific studies and those not written in English, French, Portuguese, or Spanish. The Appraisal of Indicators through Research and Evaluation (AIRE) instrument assessed methodological quality. Domain scores and an overall score were calculated using an equal-weight principle. KPIs were classified into structure, process, and outcome categories. The protocol is available at 10.17605/OSF.IO/KS2G3.

**Results:**

We included thirteen studies that collectively developed 225 KPIs. Merely five studies scored over 50% on the AIRE instrument, with domains #3 (scientific evidence) and #4 (formulation and usage) displaying low scores. Among the KPIs, 8.4% were classified as structure, 85.8% as process, and 5.8% as outcome indicators. The overall methodological quality did not exhibit a clear association with a major focus on outcomes. None of the studies provided benchmarking reference values.

**Conclusion:**

The KPIs formulated for evaluating CPS in hospital settings primarily comprised process measures, predominantly suggested by pharmacists, with inadequate evidence support, lacked piloting or validation, and consequently, were devoid of benchmarking reference values.

**Supplementary Information:**

The online version contains supplementary material available at 10.1007/s11096-024-01717-x.

## Impact statements

- This systematic review evaluated the quality of key performance indicators for clinical pharmacy through the Appraisal of Indicators through Research and Evaluation (AIRE) instrument.
- Key performance indicators for clinical pharmacy were developed with insufficient evidence support and lacked piloting or validation.
- Patients and other healthcare professionals were not included in the consensus panels that developed clinical pharmacy KPIs.
- Clinical pharmacy KPIs predominantly focus on measuring processes rather than patients' health outcomes.
- There are no benchmarking reference values available for clinical pharmacy KPIs.

## Introduction

Health care quality is defined as the extent to which health services, for individuals and populations, enhance the likelihood of desired health outcomes and align with current professional knowledge [1]. Clinical pharmacy, integral to the healthcare system, is defined by the American College of Clinical Pharmacy (ACCP) as the "area of pharmacy concerned with the science and practice of rational medication use"[2]. While implementation guidelines for clinical pharmacy services (CPS) strongly emphasise enhancing the rational and secure use of drugs, they do not specify indicators for assessing their quality [3–6].

Differing from quality indicators [7], key performance indicators (KPIs) are quantifiable measures reflecting critical success factors of an organization and are instrumental in monitoring continuous improvement in the quality of health service delivery [8]. Various procedures have been devised for KPI development, ranging from mathematical formulation [9] to the appraisal of linguistic summaries [10]. However, consensus methods involving relevant stakeholders, such as the Delphi technique, are the most employed processes for KPI development [11–15].

While pharmacists collaborate as part of a care team, this collaboration should not hinder the pharmacy profession from quantifying its direct impact on patient care [16]. Clinical pharmacy KPIs aim to encapsulate the values of CPS and demonstrate their impact on patient outcomes to stakeholders, administrators, and multidisciplinary teams. However, as of now, there are no standardized clinical pharmacy KPIs at the national or international level.

### Aim

The aim of this systematic review was to assess the quality of the studies that developed KPIs for CPS in inpatient hospital settings.

## Method

This systematic review adhered to the Cochrane methodological recommendations [17] and followed the reporting guidelines of the Preferred Reporting Items for Systematic Reviews and Meta-Analyses (PRISMA) [18]. The review protocol was published a priori on the Open Science Framework (OSF) and can be accessed at DOI: 10.17605/OSF.IO/KS2G3.

### Literature selection

In July 2023, a systematic search process was undertaken using three bibliographic sources: Web of Science, Scopus, and PubMed. Studies addressing the development, implementation, and benchmarking of KPIs in CPS for hospital inpatients were included. The search strategies are outlined in Supplementary File 1. Additionally, a grey literature search was conducted on Google Scholar to identify studies not indexed in bibliographic databases. Furthermore, a bidirectional systematic citation analysis (backward and forward) of the included studies was performed. The backward retrieval strategy involved searching the references of all included studies, while the forward process entailed searching for articles citing the included studies using the Web of Science. All records were imported into an EndNote database (Clarivate, London, UK), and duplicate records were removed.

Two reviewers independently performed the eligibility phase in two steps. First they screened potentially relevant titles and abstracts. An inter-rater agreement analysis using the prevalence-adjusted and bias-adjusted kappa (PABAK) [19] was conducted during the screening phase, considering Kappa values over 0.7 as acceptable. Discrepancies were resolved through a consensus meeting. Secondly, full-text papers not excluded during the screening phase underwent further evaluation, and studies were excluded based on the following criteria: (1) studies lacking detailed reporting of clinical pharmacy KPIs; (2) studies solely addressing KPIs related to drug- or disease-specific conditions (e.g., infections, cancer, etc.); (3) studies written in languages other than English, French, Portuguese, or Spanish; (4) articles reporting studies conducted outside the hospital inpatient environment; and (5) studies published as editorials, letters to the editor, commentaries, or narrative case reports. Multiple articles reporting the results of the same study were consolidated.

### Data extraction and quality appraisal

Articles were appraised by one researcher to extract pertinent information, including: 1) author and year of publication; 2) country where the study was conducted; 3) objectives; 4) methods employed for KPI development; 5) composition of the team responsible for KPI evaluation; 6) number of KPIs directly related to CPS; and 7) benchmarking. We decided not contacting the researchers from the different KPI developing teams and relying only in the information they had provided in their published articles.

The Appraisal of Indicators through Research and Evaluation (AIRE) instrument [20] was employed to assess the methodological quality of the studies included in this review. AIRE, a validated instrument designed for indicator quality assessment [21], was initially derived from the AGREE (Appraisal of Guidelines Through Research and Evaluation) instrument [22]. Utilized in several systematic reviews on indicator quality [23–26], the AIRE instrument comprises 20 items across four domains: "Purpose, relevance, and organizational context," "Stakeholder involvement," "Scientific evidence," and "Additional evidence, formulation, and usage." Each item presents a statement about the quality of the indicator and is scored on a 4-point scale (1 ‘totally disagree or no information provided’ to 4 ‘strongly agree’). Two authors independently applied the AIRE instrument to each included study. Subsequently, a standardized domain score was calculated per AIRE guidelines using the formula: (total score − minimum possible score)/(maximum score − minimum possible score) × 100%. Likewise, an overall standardized score was computed using the same method across all instrument items. In both cases, a higher standardized score indicates better methodological quality (range 0–100%). Studies were classified with high methodological quality in each domain if they scored ≥ 50%, corresponding to an overall “agree” or “strongly agree” [27].

### KPI categorization

The KPIs proposed in the included studies were extracted and categorized according to Donabedian’s structure, process, or outcome (SPO) paradigm [28]. Structure measures encompass various professional and organizational resources associated with care provision. Process measures pertain to the actions clinicians do to and for patients to enhance or maintain health. Outcome measures signify changes in physical status resulting from care processes, such as morbidity, mortality, and improved quality of life. Furthermore, outcome KPIs were sub-categorized based on Kozma et al.'s economic, clinical, and humanistic outcomes (ECHO) model [29]. Economic outcomes include direct, indirect, and intangible costs; clinical outcomes are defined as disease or treatment-related results; and humanistic outcomes encompass the effects of illness or treatment on a patient's quality of life and functional status. Three authors independently conducted both categorizations, and any disagreements were resolved through discussion between the authors.

## Results

**Fig. 1:**
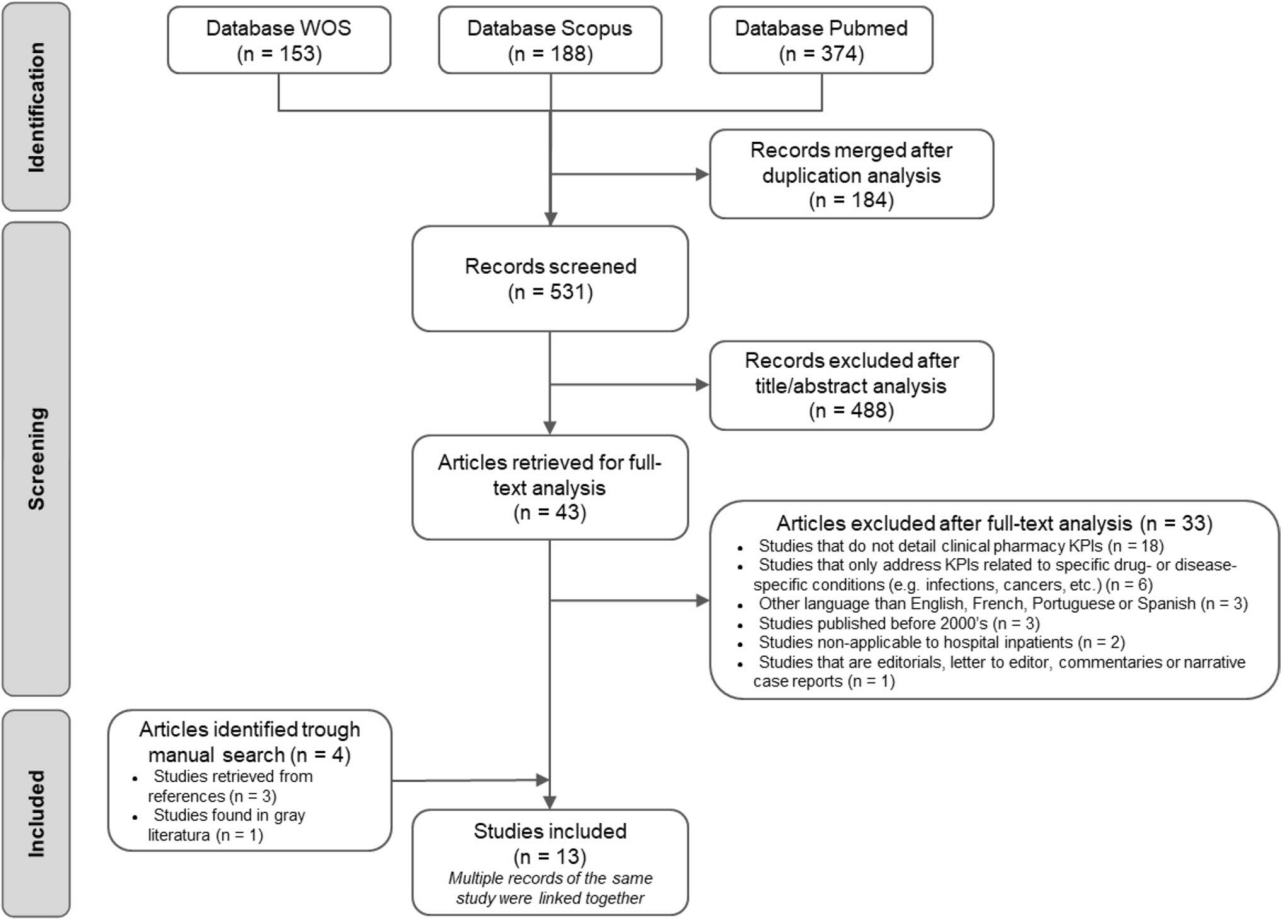
Flow diagram of the study selection process

The initial search identified 531 different records, excluding duplicates. During the screening phase, 488 records were deemed irrelevant, leaving 43 for full-text review. The inter-rater agreement between independent screeners was considered sufficient (PABAK = 0.816). Following the full-text assessment, ten articles corresponding to nine studies met the inclusion criteria and were considered for analysis. Additionally, three other articles were retrieved from citation analyses (i.e., backward and forward searches), and one was obtained from the grey literature search. Ultimately, 14 articles, corresponding to 13 studies, were included in this review (Fig. 1) [30–43].

### Study characteristics

Table 1 provides an overview of the studies included in this review. The articles, published between 2010 and 2023, are distributed across all continents: six in Europe, two in America, two in Asia, two in Oceania, and one in Africa. Notably, three studies focused on specific CPS, as declared by the authors: Doerper et al. and Aljamal et al. addressed "medication reconciliation," while King et al. concentrated on "transitions of care."

**Table 1:** Characteristics of the included studies

References	Country	Objective of study	Methodology used to develop the KPIs	Composition of the KPIs evaluation team	KPIs^a^
Ng et al. [30]	New Zeland	To identify a set of measurable KPIs demonstrating hospital clinical pharmacy’s contribution to patient care that can be used for benchmarking in the New Zealand setting	Delphi technique	Chief Medical Officer, Director of Nursing, Chief Pharmacist, Quality and Risk Manager, and senior management team member directly accountable for pharmacy services	Total: 25 - Structure: 0 - Process: 25 - Outcome: 0
Doerper et al. [31]	France	This research aimed to reorganize the medication reconciliation process to make it more efficient	Not specified	Not specified	Total: 6 - Structure: 0 - Process: 6 - Outcome: 0
Fernandes et al. [32, 33]	Canada	Develop national cpKPIs to advance clinical pharmacy practice and improve patient care	Modified Delphi technique	Hospital pharmacists, hospital pharmacy leaders, or members of national hospital pharmacy organizations	Total: 8 - Structure: 0 - Process: 8 - Outcome: 0
Aljamal et al. [34]	UK	Assess the appropriateness of the MR indicators already developed and reach a consensus on their appropriateness to assess the quality of the MR process so that they could be used to improve the MR process and the quality of patient care upon admission to the hospital	Modified Delphi technique	Pharmacists (clinical pharmacists who were practicing in UK hospitals at the time of the study, had at least three years of hospital experience, and currently were conducting MR in hospital wards)	Total: 41 - Structure: 0 - Process: 41 - Outcome: 0
Lloyd et al. [35]	Australia	To explore hospital pharmacists’ perspectives on the role of KPIs and to use their perspectives to suggest a set of KPIs for use in Australian hospital pharmacy practice	Online survey	Pharmacists	Total: 7 - Structure: 0 - Process: 7 - Outcome: 0
Cillis et al. [36]	Belgium	Develop and validate a tool for a standardized comparison of clinical pharmacy practices that could be used in Belgian hospitals. The tool aims to measure QIs and contextual factors relevant to the Belgian context	Focus groups + Delphi technique	Clinical pharmacists	Total: 10 - Structure: 0 - Process: 10 - Outcome: 0
Krzyżaniak et al. [37]	Poland	Identify a set of pharmacy-based KPIs in Donabedian’s process, structure, and outcome domains that can be used to benchmark the quality of pharmaceutical care provided to patients in Polish NICU settings. Identify the minimum level of pharmacy services that should be consistently provided to NICU patients	Delphi technique	Hospital pharmacists or pharmacists based in academia, as well as leading medical doctors and nurses, people who have experience with hospital-based clinical pharmacy services, and people with experience in the NICU	Total: 23 - Structure: 9 - Process: 11 - Outcome: 3
Shawahna et al. [38]	Palestine	Develop and achieve consensus on what activities and services to use as KPIs to capture and measure the impact of pharmacists in integrative healthcare facilities	Delphi technique	Pharmacists, physicians, nurses, risk/quality assurance managers	Total: 8 - Structure: 0 - Process: 8 - Outcome: 0
Al-Jazairi et al. [39]	Saudi Arabia	This study aimed to quantify clinical pharmacists’ contributions to patient care in a tertiary care hospital using predefined cpKPIs	Predefined cpKPIs adapted from ACCP and ASHP-PAI	Pharmacists	Total: 18 - Structure: 0 - Process: 16 - Outcome: 2
King et al. [40]	USA	The 2020 ACCP Transitions of Care Task Force was charged with updating a 2012 white paper that focused on process indicators of quality clinical pharmacy services during TOC	Literature review and Task Force updated the ACCP 2011 document	Pharmacists	Total: 16 - Structure: 0 - Process: 9 - Outcome: 7
Lopes et al. [41]	Portugal	For the first time in Portugal, define a national set of relevant and measurable cpKPIs/saKPIs to assess the National Health System Hospital Pharmacies’ performance and quality	Combined nominal and focus group technique	Pharmacists	Total: 16 - Structure: 4 - Process: 12 - Outcome: 0
Ramos et al. [42]	Spain	Develop a common framework of basic and advanced activities that guide assessing pharmaceutical care in the ED	Delphi technique + nominal group	Pharmacists and physicians	Total: 26 - Structure: 6 - Process: 20 - Outcome: 0
Anene-Okeke et al. [43]	Nigeria	To identify cpKPIs that can be used for benchmarking clinical pharmacy services provided in the Nigerian hospital setting	Delphi technique	Pharmacist	Total: 21 - Structure: 0 - Process: 20 - Outcome: 1

*AACP* American College of Clinical Pharmacy, *ASHP-PAI* American Society of Health-System Pharmacy-Practice Advancement Initiative, *cpKPI* Clinical Pharmacy Key Performance Indicator, *ED* Emergency Department, *KPI* Key Performance Indicator, *MR* Medication Reconciliation, *NICU* Neonatal Intensive Care Unit, *QI* Quality Indicator, *saKPI* Support Activity Key Performance Indicator, *TOC* Transitions of Care, *UK* United Kingdom, *USA* United States of America

*The authors referred to developed 52 KPIs, but only 25 were shown in the study

^a^KPIs were extracted and categorized for each study according to Donabedian’s structure, process, or outcome paradigm by the researchers

Most studies involved a comprehensive KPI development process, which included an appraisal of evidence from the literature, followed by an expert panel consensus. Delphi (regular or modified) was the most prevalent method for KPI development, with eight studies utilizing this approach. One study combined nominal and focus group techniques, while two studies adapted consensus indicators previously published by the American College of Clinical Pharmacy (AACP) and the American Society of Health-System Pharmacy (ASHP). Additionally, one study employed an online survey with pharmacists, and another study did not provide information regarding the KPI development approach.

Pharmacists were the predominant stakeholders in the Key Performance Indicator (KPI) development teams, represented in 12 (92%) studies. Additionally, four studies included a minority of other professionals such as physicians, nurses, and risk/quality assurance managers. One study did not provide information about the composition of stakeholders. Notably, none of the studies reported the participation of patients, payers, or policymakers.

Several studies emphasized the significance of standardizing and benchmarking KPIs. The reasons supporting this recommendation included: (1) enabling meaningful comparisons of CPS within and between organizations; (2) facilitating performance management of hospital pharmacists; and (3) enhancing transparency about the quality of hospital pharmacy services. Despite the acknowledgement of benchmark importance, none of the studies provided any.

### Critical appraisal

Table 2 presents the scores for the methodological quality of the included studies using the AIRE instrument. Notably, there is a wide variation in the information and level of detail describing the methodological characteristics of the studies developing KPIs. In domain #1 (Purpose, relevance, and organizational context), 11 studies (85%) scored above the 50% threshold, with an overall mean score of 64%. The item "the quality domain the indicator addresses is described in detail," representing the care quality aspect of the indicator (e.g., patient safety, care effectiveness, timeliness, equality, patient-centeredness, etc.), received the lowest score among the studies. In domain #2 (Stakeholder involvement), 10 studies (77%) demonstrated good quality, with an overall mean score of 54%. Two items in this domain scored particularly low: stakeholders' involvement and formal endorsement of the indicator set. Domains #3 (Scientific evidence) and #4 (Additional evidence, formulation, and usage) received the lowest scores, with overall means of 39% and 35%, respectively. Only five studies (38%) and three studies (23%) exceeded the 50% threshold in these domains. In domain #3, the item "supporting evidence has been critically appraised" scored the lowest, while in domain #4, the lowest scores were related to risk adjustment, accuracy, and consistency of the measures, as well as the piloting of the indicators.

**Table 2:** Appraisal of indicators through research and evaluation instrument score

Domains and questions/Author, year	Studies (reference numbers)													Mean score
	[30]	[31]	[32, 33]	[34]	[35]	[36]	[37]	[38]	[39]	[40]	[41]	[42]	[43]	
**Domain #1: Purpose, relevance, and organizational context (%)**	50.0	46.7	86.7	70.0	40.0	66.7	50.0	63.3	53.3	76.7	96.7	66.7	63.3	**63.8**
1.1 The purpose of the indicator is described clearly	7	3	8	6	5	7	6	8	8	7	4	4	8	
1.2 The criteria for selecting the topic of the indicator are described in detail	5	4	7	6	4	7	4	5	5	6	5	5	7	
1.3 The organizational context of the indicator is described in detail	6	7	6	6	5	5	8	5	6	7	7	7	6	
1.4 The quality domain the indicator addresses is described in detail	3	3	7	7	4	3	3	4	4	7	6	6	3	
1.5 The healthcare process covered by the indicator is described and defined in detail	4	7	8	6	4	8	4	7	3	6	8	8	5	
**Domain #2: Stakeholder involvement (%)**	61.1	5.6	55.6	38.9	44.4	72.2	61.1	55.6	66.7	66.7	61.1	61.1	50.0	**53.8**
2.1 The group developing the indicator includes individuals from relevant professional groups	8	3	6	6	7	8	8	7	6	6	7	8	7	
2.2 Considering the purpose of the indicator, all relevant stakeholders have been involved at some stage of the development process	6	2	5	3	3	5	6	5	4	5	5	5	4	
2.3 The indicator has been formally endorsed	3	2	5	4	4	6	3	4	8	7	5	4	4	
**Domain #3: Scientific evidence (%)**	16.7	0.0	77.8	27.8	22.2	61.1	50.0	33.3	55.6	72.2	38.9	33.3	22.2	**39.3**
3.1 Systematic methods were used to search for scientific evidence	3	2	7	3	4	7	6	4	6	7	5	4	3	
3.2 The indicator is based on recommendations from an evidence-based guideline or studies published in peer-reviewed scientific journals	3	2	7	5	4	7	6	5	7	7	5	5	4	
3.3 The supporting evidence has been critically appraised	3	2	6	3	2	3	3	3	3	5	3	3	3	
**Domain #4: Additional evidence, formulation and usage (%)**	24.1	53.7	35.2	31.5	35.2	50.0	29.6	24.1	55.6	25.9	33.3	29.6	31.5	**35.3**
4.1 The numerator and denominator are described in detail	2	7	4	4	4	6	5	3	5	3	6	3	5	
4.2 The target patient population of the indicator is defined clearly	3	7	4	5	5	4	7	4	4	4	4	5	4	
4.3 A strategy for risk adjustment has been considered and described	3	3	4	3	4	3	3	3	4	3	3	2	3	
4.4 The indicator measures what it is intended to measure	4	5	6	4	5	6	3	5	6	6	6	6	6	
4.5 The indicator measures accurately and consistently	3	4	4	4	4	4	3	4	3	3	3	4	4	
4.6 The indicator has sufficient discriminative power	4	3	4	4	4	5	3	4	5	4	4	4	3	
4.7 The indicator has been piloted in practice	4	7	2	3	3	6	3	2	7	4	3	3	3	
4.8 The efforts needed for data collection have been considered	4	5	4	4	5	6	3	3	7	2	3	4	4	
4.9 Specific instructions for presenting and interpreting the indicator results are provided	4	6	5	4	3	5	4	3	7	3	4	3	3	
**Overall (%)**^**a**^	**35.0**	**36.7**	**57.5**	**41.7**	**35.8**	**59.2**	**42.5**	**40.0**	**56.7**	**51.7**	**54.2**	**44.2**	**40.8**	**45.8**

^a^The overall score was not described in the original AIRE publication

Overall, two studies achieved high methodological quality in all four AIRE domains, while one study scored below the 50% threshold across all four AIRE domains. In the overall standardized score, encompassing all items from the four domains, only five studies scored higher than the 50% threshold, whereas three studies scored around 35%.

### KPI description and categorization

In total, 225 KPIs were extracted from the 13 studies. A complete list of these 225 KPI is available at Supplementary File 2. The reported number of KPIs in each study ranged from 6 to 41 (median = 16). Following Donabedian’s SPO paradigm [44], 19 KPIs (8.4%) were classified as structure, 193 (85.8%) as process, and 13 (5.8%) as outcome indicators. Among the outcome KPIs, 4 (1.8%) were economic, 7 (3.1%) were clinical, and 2 (0.9%) pertained to the humanistic dimensions of the ECHO model. Only one study developed KPIs spanning all three SPO categories. Merely three studies, contributing to 49 KPIs, described them using Donabedian’s SPO framework. Among these 49 KPIs, authors' classifications aligned with ours in 41 instances, with the remaining 8 originally classified as outcome KPIs by the authors but as process KPIs by our team. Notably, none of the studies utilized the ECHO model to categorize the KPIs. A comprehensive description of the KPIs is provided in Supplementary File 3.

Structure KPIs concentrated on the organizational aspects of the service and included supportive activities (9 KPIs in 3 studies), availability of protocol/policy (5 KPIs in 2 studies), technical and human resources (4 KPIs in 4 studies), and professional qualification (1 KPI in 1 study).

Process KPIs could be categorized into ten services: medication reconciliation (75 KPIs in 12 studies), medication review (46 KPIs in 10 studies), pharmacist advice (25 KPIs in 8 studies), care team involvement (16 KPIs in 5 studies), patient education (14 KPIs in 7 studies), care plan implementation (7 KPIs in 4 studies), dispensing (4 KPIs in 4 studies), outpatient services (2 KPIs in 2 studies), student mentoring (2 KPIs in 2 studies), and care team members’ satisfaction (2 KPIs in 1 study).

Among the 7 clinical outcomes KPIs, the focus was on adverse drug reactions (3 KPIs in 2 studies), patient readmission (3 KPIs in 1 study), and length of stay (1 KPI in 1 study). The 4 economic outcomes KPIs included cost savings (3 KPIs in 2 studies) and cost of therapy (1 KPI in 1 study). Additionally, the 2 humanistic outcomes KPIs aimed at patient satisfaction (2 KPIs in 2 studies).

A higher prevalence of outcome KPIs in a study was not associated with the overall methodological quality assessed by the AIRE. Among the five studies scoring above the 50% threshold, only two developed any outcome KPI. Notably, the study that produced KPIs from all three SPO categories scored below the 50% threshold.

## Discussion

### Statement of key findings

A total of 225 clinical pharmacy KPIs for hospital inpatient settings were identified in 13 studies conducted across all continents. The methodological quality and applicability of the KPI sets exhibited considerable variability, with a notable weakness in the supporting scientific evidence and a lack of piloting and post-development evaluation. Less than 6% of the KPIs developed in these studies were classified as outcome KPIs.

### Strengths and weaknesses

To our knowledge, this is the first systematic review focusing on KPI development studies in hospital settings. Other reviews have been conducted in different contexts [45] or have focused on specific subjects [46, 47]. Additionally, this study marks the first application of the AIRE instrument to evaluate KPIs in this setting. While other studies have applied AIRE to KPI development studies in specific conditions or activities [22, 46], the emphasis here is on generalist KPIs that are adaptable for use in different countries, facilitating international comparisons. Similarly to what happens in other assessment metrics, the debate between generalist or specific KPIs still persists [48, 49]. We preferred considering generalist KPI because they can be used in economic evaluations, allowing comparisons between CPS and any other health care technology. Although clinical pharmacy is not exclusively performed in hospital environments [50], the specificities of the hospital setting might justify the development of hospital centred KPIs to evaluate the quality of CPS.

This study has limitations. Firstly, our eligibility criteria excluded disease- or drug-specific studies, potentially limiting the variety of KPIs. Nevertheless, the generalist characteristic of the included studies can be considered a strength. Like any evidence-gathering exercise, some relevant literature may not have been identified. To mitigate this potential limitation, we conducted searches in the three most widely used bibliographic databases, performed a thorough citation analysis both backward and forward, and conducted an extensive grey literature search. And, as with any methodological quality appraisal assessment, the results of the AIRE are influenced by the comprehensiveness of the studies' reporting. Poor reporting can lead to underestimating the methodological quality of any study. Additionally, AIRE is an instrument with limited methodological guidance available, leaving room for users' interpretation [22]. Despite the general belief that AIRE domains should not be aggregated into an overall score, as no confirmatory factorial analysis evaluated its fit to a pre-established structure, we opted to create an overall score attributing equal weight to each domain. While this approach could be subject to debate, we supported it based on the same rationale that AIRE attributes equal weight to items in each domain.

### Interpretation

In general, the quality of the studies that developed KPIs for CPS in hospital setting was low, with an AIRE score of around 45%. Two items were poorly scored: evidence supporting the KPIs and the lack of piloting and post-development evaluation. Our findings align with previous analyses in areas such as school feeding programs [23], long-term care [26], or dental care [24]. Higher scores in domain #1 (Purpose, relevance, and context) suggest that KPI developers have a clear vision of their objectives. In domain #2 (Stakeholder involvement), the difference between the higher scores of items 2.1 and 2.2 may indicate that KPI developers included highly relevant stakeholders but may have overlooked some essential ones, which is consistent with a review of quality indicators for community care for older people [25]. The poor evidence supporting the developed KPIs may signify a need for more research, not only before KPI development but also during the development process.The low scores in domain #4 are associated with a poor description of the developed KPIs, likely due to inadequate research and evidence support, and the absence of a piloting exercise to validate the new indicators. This observation aligns with previous literature. [25]. In summary, it appears that KPIs are developed by highly motivated and expert steering groups, possibly working in isolation from some stakeholders. The ideas behind these KPIs may not be sufficiently supported by scientific evidence, and there is a lack of adequate validation before publishing the development report[51].

An important step in KPI development is achieving consensus to select indicators. The Delphi technique facilitates the development of quality indicators in areas where the evidence is insufficient or controversial, synthesizing accumulated expert opinion [52, 53]. A key issue to strengthen the consensus and enhance the credibility and acceptability of the agreed KPIs is ensuring the involvement of panellists including all potential stakeholders [54]. The selection of appropriate stakeholders in healthcare innovation has been demonstrated to be complex [55]. In our review, eight studies (61%) adopted the Delphi technique, but only four included professionals other than pharmacists.

Less than 6% of the compiled KPIs were classified as outcome-related KPIs, a finding consistent with other analyses of KPI development studies [46]. The limited focus on outcome KPIs may be associated with the unclear aim of clinical pharmacy towards health outcomes. The abridged definition of clinical pharmacy provided by the ACCP omits any mention of health outcomes. In contrast, their unabridged definition states that clinical pharmacy "optimizes medication therapy and promotes health, wellness, and disease prevention" [2]. While optimizing therapy is part of the care process, the remaining three goals can be considered as health outcomes. The European Society of Clinical Pharmacy's definition mentions the optimization of medicines' utilization "in order to achieve person-centred and public health goals," which are likely health outcomes [56]. Although clinical pharmacy is considered to "embrace the philosophy of pharmaceutical care," leading societies were not as explicit in outcome orientation as pharmaceutical care definitions [57, 58]. The ambiguity between these two concepts remains unresolved [50, 59].

Despite the straightforward interpretation of process indicators [46], there is a pressing need for a greater focus on health outcomes when developing KPIs for CPS. While association between process and outcome is expected, assuming a linear correlation cannot be taken for granted [28]. Salampessy et al. demonstrated that structure and process are frequently not correlated with outcome indicators [60]. The lack of clarity regarding the goals of any clinical activity, including CPS, has been criticized for fostering "gaming of the system" and impeding progress in performance improvement [61]. Process indicators have been described as having limited impact on value and garnering little attention from patients [62]. Measuring performance in healthcare should always entail measuring health outcomes. Like other healthcare areas [63], KPIs for CPS should be outcome oriented. An outcome "denotes the effects of care on the health status of patients and populations" [28]. This implies that some measures frequently described as outcomes are, in fact, process indicators (e.g., unnecessary drug use, medication complexity, medication adherence, drug-related problems) [64, 65]. This incorrect classification was common among the KPI development exercises.

Implementing KPI evaluation incurs costs that should be considered during the KPI development process. While recognizing the need for KPI assessment, clinical pharmacists have expressed concerns about the burden associated with KPI implementation [66]. In our review, the number of KPIs developed in each exercise varied from 6 to 41 (median = 16). Meyer et al. advocate for applying a parsimony principle to focus on measuring what matters, based on end-user needs [67].

### Further research

Our study revealed three major deficiencies in the clinical pharmacy KPI development processes: the absence of internationally standardized KPIs; a predominant focus on assessing processes rather than outcomes; and a complete lack of benchmarking reference values. To enhance the accountability of CPS, addressing these three deficiencies is essential. This can be achieved by creating a concise list of internationally accepted KPIs with benchmarking reference values to set minimum practice standards [68]. The use of publicly reported KPIs has been identified as a driver of higher quality levels [69].

In value-based health care systems, the assessment of performance should rely exclusively on outcome measures [62]. Outcome measures serve in cost-effectiveness analyses, demonstrating the cost–benefit ratio of CPS, but also in economic evaluations, showcasing CPS as the optimal option in certain care processes [70]. Selecting the appropriate outcomes to assess the quality of CPS poses a major challenge. The debate surrounding the validity of surrogate outcomes and hard outcomes remains unresolved in any health care field [71]. As Donabedian noted, measuring survival or mortality in non-fatal situations would not be useful [44]. Establishing a core outcome set for use in CPS KPIs should be a significant focus of clinical pharmacy research. These outcomes should be sensitive enough to possess discriminatory power when CPS are implemented and relevant enough to facilitate comparisons with other health care interventions.

## Conclusion

Our study reveals that the clinical pharmacy KPIs developed for the evaluation of CPS in hospital settings were predominantly process measures, crafted by expert panels mainly composed of pharmacists, lacking robust scientific evidence support, and were not piloted or validated, thereby lacking benchmarking reference values. Future efforts should focus on creating an internationally standardized core outcome set to effectively measure the quality of CPS and facilitate comparisons of their value with other healthcare activities.

### Supplementary Information

Below is the link to the electronic supplementary material.

Supplementary file1 (PDF 50 kb)

Supplementary file2 (PDF 327 kb)

Supplementary file3 (PDF 462 kb)
