# Supplementary material for: Clinical pharmacy key performance indicators for hospital inpatient setting: a systematic review
Source: Int J Clin Pharm. 2024 Apr 3;46(3):602–13. doi: 10.1007/s11096-024-01717-x (PMC11133179; doi:10.1007/s11096-024-01717-x)
Supplement: Supplementary file 1 — Supplementary file1 (PDF 50 kb) [file 11096_2024_1717_MOESM1_ESM.pdf]

Magedanz L, Silva HL, Galato D, Fernandez-Llimos F. Clinical Pharmacy Key Performance Indicators for Hospital Inpatient Setting: A Systematic Review

Supplementary material S1. Search strategies

Pubmed

#1: ("Key performance indicators"[TIAB] OR "KPI"[TIAB] OR "Quality indicators"[TIAB] OR "Benchmark"[TIAB] OR "Benchmarking tool"[TIAB] OR "Consensus indicators"[TIAB] OR "Performance indicators"[TIAB] OR "Quality indicators"[TIAB] OR "clinical metrics"[TIAB] OR "Standards of care"[TIAB] OR "Quality indicators, Health care"[MH] OR "Benchmarking"[MH])

#2: ("Pharmacist intervention"[TIAB] OR "Clinical Pharmacy"[TIAB] OR "Pharmaceutical care"[TIAB] OR "Pharmaceutical service"[TIAB] OR "Pharmacy service"[TIAB] OR "Pharmacy practice"[TIAB] OR "Pharmacy service, hospital"[TIAB] OR "Clinical pharmacist service"[TIAB] OR "Pharmacist"[TIAB] OR "Pharmacy Service, Hospital"[MH] OR "Pharmaceutical services"[MH] OR "Pharmacists"[MH])

#3: ("Hospital"[TIAB] OR “Hospitals”[MH] OR “Pharmacy Service, Hospital”[MH])

#1 AND #2 AND #3

Scopus

(TITLE-ABS (pharmacy OR pharmacist)) AND (TITLE-ABS (clinical OR care)) AND (TITLE-ABS (hospital)) AND ((TITLE-ABS "Key performance indicators" OR "KPI" OR "Support activities indicators" OR "Quality indicators" OR "Benchmark" OR "Benchmarking tool" OR "Consensus indicators" OR "Performance indicators" OR "clinical metrics"))

Web of Science

TS=("Key performance indicators" OR "KPI" OR "Support activities indicators" OR "Quality indicators" OR "Benchmark" OR "Benchmarking tool" OR "Consensus indicators" OR "Performance indicators" OR "Quality indicators" OR "clinical metrics") AND TS=(Hospital) AND TS=(pharmacy OR pharmacist) AND TS=(clinical OR care)
