# Supplementary material for: Clinical pharmacy key performance indicators for hospital inpatient setting: a systematic review
Source: Int J Clin Pharm. 2024 Apr 3;46(3):602–13. doi: 10.1007/s11096-024-01717-x (PMC11133179; doi:10.1007/s11096-024-01717-x)
Supplement: Supplementary file 2 — Supplementary file2 (PDF 327 kb) [file 11096_2024_1717_MOESM2_ESM.pdf]

Supplementary material S2. Clinical pharmacy KPI metadata (as provided by the developers)

Author, year	KPI Description	Clinical Pharmacy theme/activity	Metric	Activity area / Clinical pharmacy service (NC = not classified)
Al-Jazairi, 2021	Number of interventions during clinical rounds	Pharmaceutical advice	n	NC
Al-Jazairi, 2021	Percentage of accepted interventions	Pharmaceutical advice	%	NC
Al-Jazairi, 2021	Number of patients reviewed or seen at clinic	Prescription review	n	NC
Al-Jazairi, 2021	Number of medication reconciliations for new admissions	Medicine reconciliation	n	NC
Al-Jazairi, 2021	Number of pharmacokinetic consultations	Pharmaceutical advice	n	NC
Al-Jazairi, 2021	Number of total parenteral nutrition (TPN) consultations and follow-ups	Pharmaceutical advice	n	NC
Al-Jazairi, 2021	Number of medication errors reported	Prescription review	n	NC
Al-Jazairi, 2021	Number of inservices (including grand rounds and journal clubs)	Multidisciplinary team strategy	n	NC
Al-Jazairi, 2021	Number of precepted residents/students	students preception	n	NC
Al-Jazairi, 2021	Number of guidelines and protocol reviews	Multidisciplinary team strategy	n	NC
Al-Jazairi, 2021	Number of committee meetings attended	Multidisciplinary team strategy	n	NC
Al-Jazairi, 2021	Number of medication use evaluations (MUEs)	Prescription review	n	NC
Al-Jazairi, 2021	Number of verified medication orders	Prescription review	n	NC
Al-Jazairi, 2021	Number of discharge consultations	patient education	n	NC
Al-Jazairi, 2021	Incidents of participation in resolving and troubleshooting medication supply issues	dispensing issues	n	NC
Al-Jazairi, 2021	Number of unapproved indication form evaluation	Prescription review	n	NC
Al-Jazairi, 2021	Number of adverse drug reaction reported	Adverse Drug Reaction		NC
Al-Jazairi, 2021	Associated cost-savings with new formulary and non-formulary request evaluations and MUEs	cost savings		NC
Cillis, 2018	New patients admitted for whom the pharmacist checked that all stages of medication reconciliation were adequately performed within 24 working hours of admission/Number of new patients admitted	Medicine reconciliation	proportion	Medication reconciliation at admission
Cillis, 2018	Number of interventions accepted and partially or completely applied by the health care team/Total number of interventions within the health care team	Pharmaceutical advice	proportion	Monitoring
Cillis, 2018	Number of interventions accepted and partially or completely applied by the health care team/Number of patients with a pharmaceutical record	Pharmaceutical advice	proportion	Monitoring
Cillis, 2018	Number of patients with a pharmaceutical record/(Number of patients present at the beginning of recording period + Number of new patients admitted)	Prescription review	proportion	Monitoring
Cillis, 2018	Number of answers to questions from the health care team/Number of weeks	Pharmaceutical advice	proportion	Information provided to the health care team

Author, year	KPI Description	Clinical Pharmacy theme/activity	Metric	Activity area / Clinical pharmacy service (NC = not classified)
Cillis, 2018	Number of patients who received therapeutic education (apart from discharges and transfers)/(Number of patients present at the beginning of recording period + Number of new patients admitted)	patient education	proportion	Patient education
Cillis, 2018	Number of patients who have received oral information about their medication before their discharge or transfer from the service/Number of patients discharged or transferred from the service	patient education	proportion	Discharge and transfer medication counselling
Cillis, 2018	Number of patients who received written information about their medication on discharge or transfer from the service/Number of patients discharged or transferred from the service	patient education	proportion	Discharge and transfer medication counselling
Cillis, 2018	Number of GPs, specialists, and/or community pharmacists who received written information about their patient's medication on discharge from the service/Number of patients discharged or transferred from the service	Pharmaceutical advice	proportion	Discharge and transfer medication counselling
Cillis, 2018	Number of interventions accepted and activities performed to prevent, detect, assess, manage, report, and/or document adverse drug reactions/Number of patients with a pharmaceutical record	Prescription review	proportion	Adverse drug reaction monitoring
Doerper, 2013	% of patients reconciled/number of eligible patients	Medicine reconciliation	%	Conciliação
Doerper, 2013	% of patients reconciled in 24 hours/number of patients reconciled	Medicine reconciliation	%	Conciliação
Doerper, 2013	% of patients reconciled retroactively versus proactively	Medicine reconciliation	%	Conciliação
Doerper, 2013	% of patients with at least 1 corrected ME/patients reconciled retroactively	Medicine reconciliation	%	Conciliação
Doerper, 2013	Number of drug error intercepted/patient reconciled retroactively	Medicine reconciliation	proportion	Conciliação
Doerper, 2013	Number of potential drug errors intercepted/patient reconciled retroactively	Medicine reconciliation	proportion	Conciliação
Fernandes, 2015	Proportion of patients who receive formal documented discharge medication reconciliation and resolution of identified discrepancies by a pharmacist	Medicine reconciliation	proportion	Discharge medication reconciliation
Fernandes, 2015	Number (or proportion) of patients who receive formal documented admission medication reconciliation by a pharmacist (includes a pharmacist best-possible medication history or pharmacist best-possible medication history review as part of the medication reconciliation process as well as resolution of identified discrepancies)	Medicine reconciliation	n / proportion	Admission medication reconciliation and bestpossible medication history
Fernandes, 2015	Number (or proportion) of pharmacists who actively participate in interprofessional patient care rounds to improve medication management	Multidisciplinary team strategy	n / proportion	Interprofessional patient care rounds

Author, year	KPI Description	Clinical Pharmacy theme/activity	Metric	Activity area / Clinical pharmacy service (NC = not classified)
Fernandes, 2015	Number (proportion) of patients for whom clinical pharmacists have completed (executed/implemented) a pharmaceutical care plan	care plan implementation	n	Pharmaceutical care
Fernandes, 2015	Number of total drug therapy problems resolved by pharmacists	Prescription review	n	Pharmaceutical care
Fernandes, 2015	Number (or proportion) of patients receiving proactive comprehensive direct patient care by a pharmacist in collaboration with the health care team	care plan implementation	n / proportion	Bundle of cpKPI critical activity areas
Fernandes, 2015	Number (or proportion) of hospital patients who receive medication counseling by a pharmacist at discharge	patient education	n / proportion	Patient education/discharge counseling
Fernandes, 2015	Number (or proportion) of patients who have received in-person education from a pharmacist about their disease(s) and medication(s) during their hospital stay	patient education	n / proportion	Patient education/discharge counseling
King, 2021	Proportion of patients within a total target population receiving specific TOC service(s) aimed at identifying medication discrepancies and medication therapy problems	Medicine reconciliation	proportion	Transition of care
King, 2021	Medication discrepancies<br>◦ Total number and distribution, overall and by type	Medicine reconciliation	n	Transition of care
King, 2021	Medication discrepancies and medication therapy problems<br>◦ Total number and distribution, overall and by type<br>◦ Percentage of patients with ≥1, overall and by type	Medicine reconciliation	n / %	Transition of care
King, 2021	Resolution rate of medication discrepancies and medication therapy problems: total percent successfully resolved, overall and by type	Medicine reconciliation	rate	Transition of care
King, 2021	Completion rates of first planned follow-up contact within prespecified time intervals, including time until follow-up<br>◦ For example, completion of postdischarge telephone interview within 72 hours, completion of postdischarge ambulatory care visit within 7 days (primary care, specialty care, and/or other referrals)	outpatient activity	rate	Transition of care
King, 2021	Completion rates of pharmacist-referred visits for preventive medicine or other medication-related concerns	Prescription review	rate	Transition of care
King, 2021	Clinician Satisfaction (including pharmacists)<br>◦ Satisfaction of clinicians with pharmacists and specific services	team member's satisfaction	score	Transition of care
King, 2021	Clinician engagement<br>◦ Readiness of clinicians to establish new services<br>◦ Rates of clinician engagement with new or existing services	team member's satisfaction	rate	Transition of care
King, 2021	Patient engagement<br>◦ Medication adherence<br>▪ Verified prescription capture rates over time, such as through insurance claims data, including whether prescriptions were physically obtained, delivered, or administered to patients	Prescription review	score / %	Transition of care

Author, year	KPI Description	Clinical Pharmacy theme/activity	Metric	Activity area / Clinical pharmacy service (NC = not classified)
	▪ Verified medication adherence (taking >80% of prescribed doses for scheduled medications)<br>▪ Consider the percentage of days covered, percentage of patients taking >80% of prescribed doses, 8-item Morisky Medication Adherence Scale (MMAS-8), pill counts, or drug concentrations<br>◦ Patient Activation Assessment score<br>◦ Completion rates of prespecified follow-up appointments (eg, inperson, by telephone, or through telemedicine) with pharmacists or other providers			
King, 2021	Rates of unplanned 30-day, all-cause hospital readmissions	patient readmission		Transition of care
King, 2021	Rates of unplanned 30-day, all-cause hospital readmissions plus ED visits	patient readmission		Transition of care
King, 2021	Total hospital length of stay (measured as hours or days)<br>◦ ICU and non-ICU portions should be included to assess TOC interventions during TOC within the hospital	lenght of stay		Transition of care
King, 2021	Rates of unplanned Health care utilization (HCU) within various time points, including medication-related causes	patient readmission		Transition of care
King, 2021	Patient satisfaction<br>◦ HCAHPS or CAHPS scores (for Medicare and Medicaid populations)<br>▪ Specifically, with respect to survey items pertaining to medications<br>◦ Items adapted from CMS surveys or other validated surveys aimed at specific TOC services within specific TOC settings<br>◦ Press Ganey<br>◦ Care Transitions Measure	satisfaction evaluation		Transition of care
King, 2021	Return on investment (ROI): Net revenue generated OR the ratio of total cost savings to total cost of servicea<br>◦ Direct and indirect costs of providing the service<br>▪ Cost per unit of time spent by each discipline involved<br>▪ Cost of physical resources necessary to provide the service<br>▪ Cost of training, administrative responsibilities, and other overhead costs<br>◦ Direct and indirect cost savings<br>▪ Cost saved from interventions focused on minimizing institutional costs while maintaining or optimizing drug therapy<br>▪ Cost saved to institutions or payers, such as through interventions that help avoid financial penalties from excessive readmission rates, reduce hospital length of stay, or help maintain quality ratings	cost savings		Transition of care

Author, year	KPI Description	Clinical Pharmacy theme/activity	Metric	Activity area / Clinical pharmacy service (NC = not classified)
King, 2021	• Direct cost savings<br>◦ Difference between actual and expected cost for a service, medication, or other health care-related resource (eg, planned vs unplanned HCU, avoided health care costs after adverse drug reactions)	cost savings		Transition of care
Krzyżaniak, 2018	Availability of written policies/protocols/guidelines for high-risk medications i.e. antibiotics, pain-relief, parenteral nutrition	availability of protocol/policy		Facilities/environment/resources
Krzyżaniak, 2018	Availability of clear policies on how to prescribe, dispense, administer and monitor medications in the NICU	availability of protocol/policy		Facilities/environment/resources
Krzyżaniak, 2018	Availability of emergency medicines sheets, with listed doses per weight	availability of protocol/policy		Facilities/environment/resources
Krzyżaniak, 2018	Availability of standard neonatal/pediatric references for use in the selection, use and evaluation of medications i.e. textbooks (BNF P, Neofax), online resources	availability of protocol/policy		Facilities/environment/resources
Ramos, 2023	Availability of circuits and procedures for the pharmacokinetic monitoring of high-risk medications.	availability of protocol/policy		Monitorización farmacocinética
Ramos, 2023	Report antimicrobial consumption in the emergency setting.	resource (information)		Programas de optimización de uso de antimicrobianos (PROA)
Krzyżaniak, 2018	Availability of electronic medication error and adverse drug event reporting (systems)	supportive activity		Facilities/environment/resources
Lopes, 2021	Percentage of services with pharmacist rounds	supportive activity		Rounds
Lopes, 2021	Existence of specifc outpatient pharmaceutical consultations (Identify which specialties)	supportive activity		Outpatient activity
Krzyżaniak, 2018	Proportion of unlicensed/of-label prescriptions that involved the consultation of a pharmacist	Prescription review	proportion	NC
Krzyżaniak, 2018	Proportion of adverse drug events that were identifed, monitored, rectifed, prevented, and reported per number of admissions	Prescription review	proportion	NC
Krzyżaniak, 2018	Proportion of dispensing errors identifed and rectifed by pharmacist per number of admissions	dispensing issues	proportion	NC
Krzyżaniak, 2018	Number of pharmacotherapy related consultations provided to medical personnel by pharmacists	Pharmaceutical advice	n	NC
Krzyżaniak, 2018	Proportion of TPN regimens that have been monitored/optimized by a pharmacist	prescription review	proportion	NC
Krzyżaniak, 2018	Proportion of IV medications that have been monitored by a pharmacist	prescription review	proportion	NC
Krzyżaniak, 2018	Proportion of dose calculations checked by pharmacist before administration	Prescription review	proportion	NC

Author, year	KPI Description	Clinical Pharmacy theme/activity	Metric	Activity area / Clinical pharmacy service (NC = not classified)
Krzyżaniak, 2018	Proportion of patients whose therapy is being monitored by a pharmacist	prescription review	proportion	NC
Krzyżaniak, 2018	Proportion of extemporaneous medications that have been prepared and monitored by a pharmacist for the NICU	prescription review	proportion	NC
Krzyżaniak, 2018	Percentage of medication orders that include the correct dose per kilogram (or body surface area) AND an effective and safe total dose	Prescription review	%	NC
Krzyżaniak, 2018	Medication error rates/reports per 6 months	Prescription review	rate	NC
Krzyżaniak, 2018	Monthly audit of episodes of antibiotic-associated adverse events	Adverse Drug Reaction		NC
Krzyżaniak, 2018	Adverse drug event rates/reports per 6 months	Adverse Drug Reaction		NC
Krzyżaniak, 2018	Costs of therapy	cost of therapy		NC
Lopes, 2021	Existence of written information regarding prescribed medications at discharge (yes / no)	supportive activity		Information sharing
Lopes, 2021	Existence of written information regarding outpatients prescribed medications (yes / no)	supportive activity		Information sharing
Ramos, 2023	Availability of a conciliation program in the emergency department.	supportive activity		Revisión/Validación de prescripciones
Ramos, 2023	High conciliation program available.	supportive activity		Revisión/Validación de prescripciones
Lopes, 2021	Number of inpatients with therapeutic reconciliation, adjusted by pharmacist FTE	Medicine reconciliation	n	Prescription review and reconciliation
Lopes, 2021	Number of inpatient prescriptions validations (medication review), adjusted by pharmacist FTE	prescription review	n	Prescription review and reconciliation
Lopes, 2021	Existence of medication reconciliations up to 72 h after admission (yes / no)	Medicine reconciliation	proportion	Prescription review and reconciliation
Lopes, 2021	Existence of medication reconciliations at discharge (Yes / No)	Medicine reconciliation	proportion	Prescription review and reconciliation
Lopes, 2021	Number of outpatient prescription validations (medication review), adjusted by pharmacist FTE	prescription review	n	Prescription review and reconciliation
Lopes, 2021	Number of pharmacist interventions in patient therapy, adjusted by pharmacist FTE	Pharmaceutical advice	n	Prescription review and reconciliation
Lopes, 2021	Number of blood products orders analysed, per 1000 patients discharged	prescription review	n	Prescription review and reconciliation
Lopes, 2021	Number of blood products orders dispensed, per 1000 patients discharged	dispensing issues	n	Prescription review and reconciliation
Lopes, 2021	Number of blood products orders analyzed, per 1000 patients discharged	prescription review	n	Prescription review and reconciliation
Lopes, 2021	Number of narcotic and psychotropic requests analysed, per 1000 patients discharged	prescription review	n	Prescription review and reconciliation
Lopes, 2021	Number of narcotic and psychotropic requests dispensed, per 1000 patients discharged	dispensing issues	n	Prescription review and reconciliation

Author, year	KPI Description	Clinical Pharmacy theme/activity	Metric	Activity area / Clinical pharmacy service (NC = not classified)
Lopes, 2021	Number of outpatient pharmaceutical consultations, adjusted by pharmacist FTE	outpatient activity	n	Outpatient activity
Ng, 2010	KPI02 Medication reconciliation: Proportion of patients for whom medication reconciliation is undertaken and discrepancies identified are resolved	Medicine reconciliation	proportion	Medication reconciliation
Ng, 2010	KPI45 Proportion of patients with a toxic or sub-therapeutic aminoglycoside concentration whose dosage has been adjusted or reviewed prior to the next dose	Prescription review	proportion	NC
Ng, 2010	KPI27 Proportion of paediatric medication orders that include the correct dose per kilogram (or body surface area) AND a safe total dose	Prescription review	proportion	NC
Ng, 2010	KPI05 Proportion of patients discharged on warfarin that receive written information regarding warfarin management prior to discharge	patient education	proportion	NC
Ng, 2010	KPI21 Chart review: Proportion of medicine charts reviewed by clinical pharmacists within 24 hours of admission	medicine reconciliation	proportion	Chart review
Ng, 2010	KPI42 Proportion of patients with an INR above 4 whose dosage has been adjusted or reviewed prior to the next warfarin dose	Prescription review	proportion	NC
Ng, 2010	KPI30 Proportion of patients receiving appropriate initial antibiotic selection for CAP (Community acquired pneumonia)	Prescription review	proportion	NC
Ng, 2010	KPI35 Proportion of patients undergoing specified surgical procedures that receive an appropriate prophylactic antibiotic regimen	Prescription review	proportion	NC
Ng, 2010	KPI28 Proportion of medication orders for intermittent therapy that are prescribed safely (e.g. alternate days or once weekly regimen)	Prescription review	proportion	NC
Ng, 2010	KPI34 Proportion of patients at high risk of venous thromboembolism that receive appropriate prophylaxis	Prescription review	proportion	NC
Ng, 2010	KPI01 Accurate medication history: Proportion of patients with an accurate documented record of their medication taking behaviour	medicine reconciliation	proportion	NC
Ng, 2010	KPI15 Chemotherapy: Proportions of patients receiving cytotoxic chemotherapy whose treatment is guided by a hospital approved chemotherapy treatment protocol	Prescription review	proportion	NC
Ng, 2010	KPI16 Proportion of patients prescribed hospital initiated warfarin whose loading doses are consistent with a hospital approved protocol	Prescription review	proportion	NC
Ng, 2010	KPI17 Proportion of patients presenting with community acquired pneumonia that are prescribed guideline concordant antibiotic therapy	Prescription review	proportion	NC

Author, year	KPI Description	Clinical Pharmacy theme/activity	Metric	Activity area / Clinical pharmacy service (NC = not classified)
Ng, 2010	KPI18 Proportion of prescriptions for restricted antibiotics that are concordant with hospital approved criteria	Prescription review	proportion	NC
Ng, 2010	KPI22 Proportion of patients that are reviewed by a clinical pharmacist within 24 hours of admission	medicine reconciliation	proportion	NC
Ng, 2010	KPI23 Pharmaceutical care plan: Proportion of patients where clinical pharmacists have completed a documented comprehensive care plan on the medicines regimen	care plan implementation	proportion	NC
Ng, 2010	KPI24 Clinical pharmacy interventions: Identification and resolution of potential or actual drug related problems per patient bed day	pharmaceutical advice	rate	NC
Ng, 2010	KPI25 Prescribing errors: Identification and resolution of unintentional departure from recommended prescribing practices per patient bed day	Prescription review	rate	NC
Ng, 2010	KPI37 Documented allergy status: Proportion of patients with incomplete allergy status at any point	medicine reconciliation	proportion	NC
Ng, 2010	KPI38 Patient counselling: Proportion of patients who have had a face-to-face discussion about medicines-specific information	patient education	proportion	NC
Ng, 2010	KPI39 Medication card provision: Proportion of patients provided with a medicines information card containing the patient's current medicines	patient education	proportion	NC
Ng, 2010	KPI40 Discharge counselling: Proportion of patients that have had a formal discussion about their medicines immediately prior to discharge	patient education	proportion	NC
Ng, 2010	KPI41 Administration errors: Identification and resolution of unintended departure from recommended administration practices per patient bed day	Prescription review	rate	NC
Ng, 2010	KPI43 Adverse drug reaction monitoring: Prevention, detection, assessment, management, appropriate reporting and documentation of adverse drug reactions per number of admissions	Prescription review	proportion	NC
Ramos, 2023	Medication reconciliation program/protocol in the emergency department.	supportive activity		Revisión/Validación de prescripciones
Ramos, 2023	Participation in programs optimization programs for the use of antimicrobials in the Emergency Department	supportive activity		Programas de optimización de uso de antimicrobianos (PROA)
Krzyżaniak, 2018	Availability of suitable fridges for vaccines and TPN on the ward	resource (equipment)		Facilities/environment/resources
Krzyżaniak, 2018	Direct availability on the ward of essential medicines for specific use within the NICU	resource (material)		Facilities/environment/resources
Krzyżaniak, 2018	Availability of a funded NICU clinical pharmacist position (full-time/part-time) in the hospital	resource (pharmacist)		Personel

Author, year	KPI Description	Clinical Pharmacy theme/activity	Metric	Activity area / Clinical pharmacy service (NC = not classified)
Krzyżaniak, 2018	NICU pharmacist holds qualifications in clinical pharmacy or NICU/pediatric pharmacy	qualification		Personel
Ramos, 2023	Number of proposed interventions.	Pharmaceutical advice	n	Revisión/Validación de prescripciones
Ramos, 2023	% validation in electronic prescription program.	Prescription review	%	Revisión/Validación de prescripciones
Ramos, 2023	% acceptance of proposed interventions.	Pharmaceutical advice	%	Revisión/Validación de prescripciones
Ramos, 2023	Number of consultations received.	Pharmaceutical advice	n	Revisión/Validación de prescripciones
Ramos, 2023	Number of medications reconciled.	Medicine reconciliation	n	Revisión/Validación de prescripciones
Ramos, 2023	Number of modified discharge prescriptions.	Pharmaceutical advice	n	Revisión/Validación de prescripciones
Ramos, 2023	Number of modified discharge prescriptions.	Pharmaceutical advice	n	Revisión/Validación de prescripciones
Ramos, 2023	Validation percentage upon admission and discharge.	Medicine reconciliation	%	Revisión/Validación de prescripciones
Ramos, 2023	% Number of justified discrepancies detected.	Medicine reconciliation	%	Revisión/Validación de prescripciones
Ramos, 2023	Number of pharmacokinetic recommendations made.	Pharmaceutical advice	n	Monitorización farmacocinética
Ramos, 2023	Participation in clinical rounds of patient evaluation.	Multidisciplinary team strategy	proportion	Participación rondas clínicas
Ramos, 2023	Participation in pain management adaptation programs.	Multidisciplinary team strategy	proportion	Adecuación manejo del dolor
Ramos, 2023	Participation in sepsis code codes/protocols.	Multidisciplinary team strategy	proportion	Código sepsis
Ramos, 2023	Number of interventions performed in sepsis code.	Pharmaceutical advice	n	Código sepsis
Ramos, 2023	Participation in cardiopulmonary resuscitation codes/protocols.	Multidisciplinary team strategy	proportion	Resucitación cardiopulmonar/Código Infarto
Ramos, 2023	Number of interventions performed in heart attack code.	Pharmaceutical advice	n	Resucitación cardiopulmonar/Código Infarto
Ramos, 2023	Participation in stroke code codes/protocols.	Multidisciplinary team strategy	proportion	Código ictus
Ramos, 2023	Number of interventions performed in stroke code.	Pharmaceutical advice	n	Código ictus
Ramos, 2023	Participation in polytrauma code codes/protocols.	Multidisciplinary team strategy	proportion	Código politrauma
Ramos, 2023	Number of interventions performed in polytrauma code.	Pharmaceutical advice	n	Código politrauma
Anene-Okeke, 2022	Number of patients who receive formal documented admission medication reconciliation by a pharmacist	Medicine reconciliation	n	NC
Anene-Okeke, 2022	Number of pharmacists who actively participate in inter professional patient care rounds to improve medication management	care plan implementation	n	NC
Anene-Okeke, 2022	Number of patients for whom clinical pharmacists have completed (executed/implemented) a pharmaceutical care plan	care plan implementation	n	NC
Anene-Okeke, 2022	Number of total drug therapy problems resolved by pharmacists	Pharmaceutical advice	n	NC
Anene-Okeke, 2022	Number of patients receiving comprehensive direct patient care by a pharmacist in collaboration with the health care team.	Multidisciplinary team strategy	n	NC
Anene-Okeke, 2022	Proportion of patients who receive formal documented discharge medication reconciliation and resolution of identified discrepancies by a pharmacist	Medicine reconciliation	proportion	NC
Anene-Okeke, 2022	Number of hospital patients who receive medication counselling by a pharmacist at discharge	patient education	n	NC
Anene-Okeke, 2022	Number of patients who have received education from a pharmacist about their disease(s) and medication(s) during their hospital stay	patient education	n	NC

Author, year	KPI Description	Clinical Pharmacy theme/activity	Metric	Activity area / Clinical pharmacy service (NC = not classified)
Anene-Okeke, 2022	Proportion of medicine charts reviewed by clinical pharmacists within 24 hours of admission	prescription review	proportion	NC
Anene-Okeke, 2022	Number of patients who have a complete and accurate list of their current medications (including over the counter and complementary medications) documented	Medicine reconciliation	n	NC
Anene-Okeke, 2022	The number of drug information enquiries that have been answered	Pharmaceutical advice	n	NC
Anene-Okeke, 2022	Number of attempted clinical interventions by the pharmacists that were accepted by the clinician	Pharmaceutical advice	n	NC
Anene-Okeke, 2022	The number of ward meetings attended by the pharmacist	Multidisciplinary team strategy	n	NC
Anene-Okeke, 2022	Total number of patients reviewed by clinical pharmacy services per month	prescription review	n	NC
Anene-Okeke, 2022	Number of medication errors reported	Prescription review	n	NC
Anene-Okeke, 2022	Number of instances of in-service education (Journal clubs, Staff education)	Multidisciplinary team strategy	n	NC
Anene-Okeke, 2022	Number of students/ Residents perecepted	students preception	proportion	NC
Anene-Okeke, 2022	Number of reviews for guidelines and protocols	Multidisciplinary team strategy	n	NC
Anene-Okeke, 2022	Number of patients who have a correctly completed record (medication and reaction) of prior Adverse Drug Reaction (ADR) and allergy documented	Prescription review	n	NC
Anene-Okeke, 2022	Prescribing errors: Identification and resolution of unintentional departure from recommended prescribing practices	Prescription review	proportion	NC
Anene-Okeke, 2022	The number of complaints that pharmacy department has received	satisfaction evaluation		NC
Aljamal, 2016	Number of patients whose drug history was compared for accuracy (correctness and completeness) with drugs prescribed on admission as a percentage of the number of patients reconciled over a defined period of time.	Medicine reconciliation	%	Medication reconciliation
Aljamal, 2016	Number of patients whose drug allergies were checked as percentage of the number of patients reconciled over a defined period of time.	Medicine reconciliation	%	Medication reconciliation
Aljamal, 2016	Number of patients for whom the names of all drugs to which they were allergic was documented as percentage of the number of patients reconciled with identified allergies over a defined period of time.	Medicine reconciliation	%	Medication reconciliation
Aljamal, 2016	Number of patients admitted to hospital whose medicines were reconciled within 24 h as a percentage of the number of patients admitted over a defined period of time.	Medicine reconciliation	%	Medication reconciliation

Author, year	KPI Description	Clinical Pharmacy theme/activity	Metric	Activity area / Clinical pharmacy service (NC = not classified)
Aljamal, 2016	Number of patients whose medication history was taken on admission (by pharmacy staff) as a percentage of the number of patients admitted over a defined period of time.	Medicine reconciliation	%	Medication reconciliation
Aljamal, 2016	Number of patients whose medication history was completed within 24 h (by pharmacy staff) as a percentage of the number of patients admitted over a defined period of time.	Medicine reconciliation	%	Medication reconciliation
Aljamal, 2016	Number of patients' drug histories that were checked using more than one source (including patient and/or GP sources) as a percentage of the number of patients reconciled over a defined period of time.	Medicine reconciliation	%	Medication reconciliation
Aljamal, 2016	Number of patients for whom a drug allergy reaction was documented as a percentage of the number of patients with identified allergies reconciled over a defined period of time.	Medicine reconciliation	%	Medication reconciliation
Aljamal, 2016	Number of patients for whom medication discrepancies were identified as a percentage of the number of patients reconciled over a defined period of time.	Medicine reconciliation	%	Medication reconciliation
Aljamal, 2016	Number of patients whose unintentional discrepancies were identified (involving strength, dose and frequency) as a percentage of the number of patients reconciled over a defined period of time.	Medicine reconciliation	%	Medication reconciliation
Aljamal, 2016	Number of total discrepancies identified per 100 admissions reconciled.	Medicine reconciliation	%	Medication reconciliation
Aljamal, 2016	Number of unintentional discrepancies identified per 100 admissions reconciled.	Medicine reconciliation	%	Medication reconciliation
Aljamal, 2016	Number of unintentional discrepancies that reached patients (identified by checking the nurse's signature on the drug chart) as a percentage of the number of unintentional discrepancies over a defined period of time.	Medicine reconciliation	%	Medication reconciliation
Aljamal, 2016	Number of patients whose medication reconciliation process was documented (using any form of documentation) as a percentage of the number of patients reconciled over a defined period of time.	Medicine reconciliation	%	Medication reconciliation
Aljamal, 2016	Number of patients whose completed medication reconciliation information was available to staff taking care of them (doctors, nurses and pharmacy staff) as a percentage of the number of patients reconciled over a defined period of time.	Medicine reconciliation	%	Medication reconciliation
Aljamal, 2016	Number of patients admitted to hospital whose medicines are reconciled within 48 h as a percentage of the number of patients admitted over a defined period of time.	Medicine reconciliation	%	Medication reconciliation

Author, year	KPI Description	Clinical Pharmacy theme/activity	Metric	Activity area / Clinical pharmacy service (NC = not classified)
Aljamal, 2016	Number of patients admitted to hospital whose medicines are reconciled within 72 h as a percentage of the number of patients admitted over a defined period of time.	Medicine reconciliation	%	Medication reconciliation
Aljamal, 2016	Number of patients for whom the medication reconciliation process was not performed as a percentage of the number of patients admitted over a defined period of time.	Medicine reconciliation	%	Medication reconciliation
Aljamal, 2016	Number of patients for whom the reasons for not performing medication reconciliation were identified (documented) as a percentage of the number of patients who had not received medication reconciliation at admission over a defined period of time.	Medicine reconciliation	%	Medication reconciliation
Aljamal, 2016	The time (in minutes) taken for completing the medication reconciliation process for patients reconciled over a defined period of time (total time required for medication reconciliation divided by number of patients reconciled).	Medicine reconciliation	time	Medication reconciliation
Aljamal, 2016	Number of patients whose drug history was performed by contacting the GP (doctor or receptionist) via phone to receive a drug history as a percentage of the number of patients reconciled over a defined period of time.	Medicine reconciliation	%	Medication reconciliation
Aljamal, 2016	Number of patients whose drug history was performed using a recent GP letter and/or repeat prescription for current admission (within one month) as a percentage of the number of patients reconciled over a defined period of time.	Medicine reconciliation	%	Medication reconciliation
Aljamal, 2016	Number of patients who were interviewed to verify their drug history as a percentage of the number of patients reconciled over a defined period of time.	Medicine reconciliation	%	Medication reconciliation
Aljamal, 2016	Number of patients whose drug history was performed using patient's own drugs (PODs) brought into hospital as a percentage of the number of patients who brought their own medication on admission over a defined period of time.	Medicine reconciliation	%	Medication reconciliation
Aljamal, 2016	Number of patients whose drug histories involved checking their history of OTC medication use (pharmacy item or general sales list) as a percentage of the number of patients reconciled over a defined period of time.	Medicine reconciliation	%	Medication reconciliation
Aljamal, 2016	Number of patients whose drug histories involved checking for history of the use of complementary (herbal) medicines as a percentage of the number of patients reconciled over a defined period of time.	Medicine reconciliation	%	Medication reconciliation

Author, year	KPI Description	Clinical Pharmacy theme/activity	Metric	Activity area / Clinical pharmacy service (NC = not classified)
Aljamal, 2016	Number of patients whose medication adherence was checked (via the patient, carer, relative or compliance aid, e.g. Venalink) as a percentage of the number of patients reconciled over a defined period of time.	Medicine reconciliation	%	Medication reconciliation
Aljamal, 2016	Number of patients for whom information about their medication adherence was communicated to the prescriber as a percentage of the number of patients who had adherence issues over a defined period of time.	Medicine reconciliation	%	Medication reconciliation
Aljamal, 2016	Number of drug history lists that included drug intolerance as a percentage of the number of drug history lists for patients reconciled over a defined period of time.	Medicine reconciliation	%	Medication reconciliation
Aljamal, 2016	Number of unintentional discrepancies involving medication omitted from the admission prescription as a percentage of the number of unintentional discrepancies identified as part of the medication reconciliation process over a defined period of time.	Medicine reconciliation	%	Medication reconciliation
Aljamal, 2016	Number of unintentional discrepancies involving the addition of a medication to the admission prescription as a percentage of the number of unintentional discrepancies identified as part of the medication reconciliation process over a defined period of time.	Medicine reconciliation	%	Medication reconciliation
Aljamal, 2016	Number of unintentional discrepancies involving a dose change in the admission prescription as a percentage of the number of unintentional discrepancies identified as part of the medication reconciliation process over a defined period of time.	Medicine reconciliation	%	Medication reconciliation
Aljamal, 2016	Number of times that unintentional discrepancies were changed (by pharmacy staff if authorised) in the medication chart as a percentage of the number of unintentional discrepancies over a defined period of time.	Medicine reconciliation	%	Medication reconciliation
Aljamal, 2016	Number of times that unintentional discrepancies were documented in the patient's medical record as a percentage of the number of unintentional discrepancies identified over a defined period of time.	Medicine reconciliation	%	Medication reconciliation
Aljamal, 2016	Number of patients whose drug history was performed by contacting the GP using fax to receive a drug history (or GP's electronic record) as a percentage of the number of patients reconciled over a defined period of time.	Medicine reconciliation	%	Medication reconciliation
Aljamal, 2016	Number of patients' carers or family members interviewed to check (clarify) drug history as a percentage of the number of patients who	Medicine reconciliation	%	Medication reconciliation

Author, year	KPI Description	Clinical Pharmacy theme/activity	Metric	Activity area / Clinical pharmacy service (NC = not classified)
	could not be communicated with and who had a contactable carer or family member over a defined period of time.			
Aljamal, 2016	Number of patients whose drug history was performed using any of the updated hospital sources (such as clinical notes of previous admissions, discharges, allergy data, or previous letters with documenting the date of the last update) as a percentage of the number of patients reconciled over a defined period of time.	Medicine reconciliation	%	Medication reconciliation
Aljamal, 2016	Number of patients whose drug history was performed using community pharmacy patient medication records (such as the Venalink compliance aid system) as a percentage of the number of patients reconciled over a defined period of time.	Medicine reconciliation	%	Medication reconciliation
Aljamal, 2016	Number of patients' drug history lists (obtained by pharmacy staff) that contained all information on medicines (drug name, strength, dose, frequency and route of administration) as a percentage of the number of drug history lists for patients reconciled over a defined period of time.	Medicine reconciliation	%	Medication reconciliation
Aljamal, 2016	Number of times that the prescriber was contacted to clarify discrepancies as a percentage of the number of patients with unintentional discrepancies identified over a defined period of time.	Medicine reconciliation	%	Medication reconciliation
Aljamal, 2016	Number of patients whose drug histories were compared with the admission prescriptions (by pharmacist) to check discrepancies as a percentage of the number of patients reconciled over a defined period of time.	Medicine reconciliation	%	Medication reconciliation
Lloyd, 2016	percentage of inpatients that have a correctly completed record (medication and reaction) of prior ADR and allergy documented within a day of admission	Medicine reconciliation	%	NC
Lloyd, 2016	percentage of hospital inpatients who receive verbal counselling and/or written information about their medicines prior to discharge	patient education	%	NC
Lloyd, 2016	percentage of discharge prescriptions reviewed and reconciled by a pharmacist prior to dispensing	Medicine reconciliation	%	NC
Lloyd, 2016	percentage of patients who have a complete and accurate list of their current medications (including over the counter and complementary medications) documented and verified within a day of admission	Medicine reconciliation	%	NC
Lloyd, 2016	percentage of discharge summaries that document an accurate medication list and the reasons for all medication therapy changes from medicines taken prior to admission	Medicine reconciliation	%	NC

Author, year	KPI Description	Clinical Pharmacy theme/activity	Metric	Activity area / Clinical pharmacy service (NC = not classified)
Lloyd, 2016	number of clinical interventions (any action by a pharmacist that directly results in a change in patient management or therapy) performed per patient bed day	Pharmaceutical advice	rate	NC
Lloyd, 2016	proportion of medicine charts reviewed by clinical pharmacists within 24 h of admission.	medicine reconciliation	proportion	NC
Shawahna, 2020	Number of medication and/or CAM (complementary and alternative medicine) related problems identified and addressed/resolved by pharmacists including contraindications, inappropriate doses (over- and/or underdoses), allergies, interactions, duplications, omissions, vague/ambiguous orders, inappropriate routes of administration, inappropriate duration of therapy, and reported ineffective therapies.	Prescription review	n	Care
Shawahna, 2020	Number of patients who received documented medication and/or CAM reconciliation by pharmacists including the best possible medication/CAM history/review and/or had their medication and/or CAM-related problems and discrepancies identified and addressed/resolved.	Medicine reconciliation	n	Reconciliation
Shawahna, 2020	Number of patients who received direct, comprehensive, and/or collaborative care by pharmacists.	care plan implementation	n	Care
Shawahna, 2020	Number of patients for whom pharmacists were involved in planning/preparing/implementing/executing/completing a therapeutic plan.	care plan implementation	n	Care
Shawahna, 2020	Number of patients who received formal counseling/education on their diseases and/or medications/CAM by pharmacists at the time of admission, stay, transition of care, and/or discharge from the healthcare facility.	patient education	n	Counseling/ education
Shawahna, 2020	Number of written complaints on the services delivered by pharmacists received per a predefined period of time.	Multidisciplinary team strategy	n	Competence/ performance/ satisfaction
Shawahna, 2020	Number of errors committed by pharmacists per a predefined period of time.	Prescription review	n	Competence/ performance/ satisfaction
Shawahna, 2020	Number of multi-healthcare provider discussions/deliberations for the purpose of improving care of patients in which pharmacists actively participated and contributed including answering formal inquiries by other healthcare providers.	Multidisciplinary team strategy	n	Multi-healthcare provider patient care
