# Supplementary material for: Clinical pharmacy key performance indicators for hospital inpatient setting: a systematic review
Source: Int J Clin Pharm. 2024 Apr 3;46(3):602–13. doi: 10.1007/s11096-024-01717-x (PMC11133179; doi:10.1007/s11096-024-01717-x)
Supplement: Supplementary file 3 — Supplementary file3 (PDF 462 kb) [file 11096_2024_1717_MOESM3_ESM.pdf]

Magedanz L, Silva HL, Galato D, Fernandez-Llimos F. Clinical Pharmacy Key Performance Indicators for Hospital Inpatient Setting: A Systematic Review

Supplementary material S3. Clinical pharmacy KPI classification

KPI classification				Original author classification	Review classification	
Author, year	Year published	Periodic	KPI Description	Type (Donabedian): • Structure (S) • Process (P) • Outcome (O) (NC = not classified)	Donabedian framework: • Structure (S) • Process (P) • Outcome (O)	Outcome classification (Kozma framework): - Economic (E) - Clinical (C) - Humanistic (H)
Al-Jazairi, 2021	2021	Hosp Pharm	Number of interventions during clinical rounds	NC	Process	
Al-Jazairi, 2021	2021	Hosp Pharm	Percentage of accepted interventions	NC	Process	
Al-Jazairi, 2021	2021	Hosp Pharm	Number of patients reviewed or seen at clinic	NC	Process	
Al-Jazairi, 2021	2021	Hosp Pharm	Number of medication reconciliations for new admissions	NC	Process	
Al-Jazairi, 2021	2021	Hosp Pharm	Number of pharmacokinetic consultations	NC	Process	
Al-Jazairi, 2021	2021	Hosp Pharm	Number of total parenteral nutrition (TPN) consultations and follow-ups	NC	Process	
Al-Jazairi, 2021	2021	Hosp Pharm	Number of medication errors reported	NC	Process	
Al-Jazairi, 2021	2021	Hosp Pharm	Number of inservices (including grand rounds and journal clubs)	NC	Process	
Al-Jazairi, 2021	2021	Hosp Pharm	Number of precepted residents/students	NC	Process	
Al-Jazairi, 2021	2021	Hosp Pharm	Number of guidelines and protocol reviews	NC	Process	
Al-Jazairi, 2021	2021	Hosp Pharm	Number of committee meetings attended	NC	Process	
Al-Jazairi, 2021	2021	Hosp Pharm	Number of medication use evaluations (MUEs)	NC	Process	
Al-Jazairi, 2021	2021	Hosp Pharm	Number of verified medication orders	NC	Process	
Al-Jazairi, 2021	2021	Hosp Pharm	Number of discharge consultations	NC	Process	
Al-Jazairi, 2021	2021	Hosp Pharm	Incidents of participation in resolving and troubleshooting medication supply issues	NC	Process	
Al-Jazairi, 2021	2021	Hosp Pharm	Number of unapproved indication form evaluation	NC	Process	
Al-Jazairi, 2021	2021	Hosp Pharm	Number of adverse drug reaction reported	NC	Outcome	Clinical
Al-Jazairi, 2021	2021	Hosp Pharm	Associated cost-savings with new formulary and non-formulary request evaluations and MUEs	NC	Outcome	Economic
Cillis, 2018	2018	Int J Clin Pharm	New patients admitted for whom the pharmacist checked that all stages of medication reconciliation were	Process	Process	

KPI classification				Original author classification	Review classification	
Author, year	Year published	Periodic	KPI Description	Type (Donabedian): • Structure (S) • Process (P) • Outcome (O) (NC = not classified)	Donabedian framework: • Structure (S) • Process (P) • Outcome (O)	Outcome classification (Kozma framework): - Economic (E) - Clinical (C) - Humanistic (H)
			adequately performed within 24 working hours of admission/Number of new patients admitted			
Cillis, 2018	2018	Int J Clin Pharm	Number of interventions accepted and partially or completely applied by the health care team/Total number of interventions within the health care team	Process	Process	
Cillis, 2018	2018	Int J Clin Pharm	Number of interventions accepted and partially or completely applied by the health care team/Number of patients with a pharmaceutical record	Process	Process	
Cillis, 2018	2018	Int J Clin Pharm	Number of patients with a pharmaceutical record/(Number of patients present at the beginning of recording period + Number of new patients admitted)	Process	Process	
Cillis, 2018	2018	Int J Clin Pharm	Number of answers to questions from the health care team/Number of weeks	Process	Process	
Cillis, 2018	2018	Int J Clin Pharm	Number of patients who received therapeutic education (apart from discharges and transfers)/(Number of patients present at the beginning of recording period + Number of new patients admitted)	Process	Process	
Cillis, 2018	2018	Int J Clin Pharm	Number of patients who have received oral information about their medication before their discharge or transfer from the service/Number of patients discharged or transferred from the service	Process	Process	
Cillis, 2018	2018	Int J Clin Pharm	Number of patients who received written information about their medication on discharge or transfer from the service/Number of patients discharged or transferred from the service	Process	Process	
Cillis, 2018	2018	Int J Clin Pharm	Number of GPs, specialists, and/or community pharmacists who received written information about their patient's medication on discharge from the	Process	Process	

KPI classification				Original author classification	Review classification	
Author, year	Year published	Periodic	KPI Description	Type (Donabedian): • Structure (S) • Process (P) • Outcome (O) (NC = not classified)	Donabedian framework: • Structure (S) • Process (P) • Outcome (O)	Outcome classification (Kozma framework): - Economic (E) - Clinical (C) - Humanistic (H)
			service/Number of patients discharged or transferred from the service			
Cillis, 2018	2018	Int J Clin Pharm	Number of interventions accepted and activities performed to prevent, detect, assess, manage, report, and/or document adverse drug reactions/Number of patients with a pharmaceutical record	Process	Process	
Doerper, 2013	2013	Pharmacien Hospitalier et Clinicien	% of patients reconciled/number of eligible patients	NC	Process	
Doerper, 2013	2013	Pharmacien Hospitalier et Clinicien	% of patients reconciled in 24 hours/number of patients reconciled	NC	Process	
Doerper, 2013	2013	Pharmacien Hospitalier et Clinicien	% of patients reconciled retroactively versus proactively	NC	Process	
Doerper, 2013	2013	Pharmacien Hospitalier et Clinicien	% of patients with at least 1 corrected ME/patients reconciled retroactively	NC	Process	
Doerper, 2013	2013	Pharmacien Hospitalier et Clinicien	Number of drug error intercepted/patient reconciled retroactively	NC	Process	
Doerper, 2013	2013	Pharmacien Hospitalier et Clinicien	Number of potential drug errors intercepted/patient reconciled retroactively	NC	Process	
Fernandes, 2015	2015	Ann Pharmacother	Proportion of patients who receive formal documented discharge medication reconciliation and resolution of identified discrepancies by a pharmacist	NC	Process	

KPI classification				Original author classification	Review classification	
Author, year	Year published	Periodic	KPI Description	Type (Donabedian): • Structure (S) • Process (P) • Outcome (O) (NC = not classified)	Donabedian framework: • Structure (S) • Process (P) • Outcome (O)	Outcome classification (Kozma framework): - Economic (E) - Clinical (C) - Humanistic (H)
Fernandes, 2015	2015	Ann Pharmacother	Number (or proportion) of patients who receive formal documented admission medication reconciliation by a pharmacist (includes a pharmacist best-possible medication history or pharmacist best-possible medication history review as part of the medication reconciliation process as well as resolution of identified discrepancies)	NC	Process	
Fernandes, 2015	2015	Ann Pharmacother	Number (or proportion) of pharmacists who actively participate in interprofessional patient care rounds to improve medication management	NC	Process	
Fernandes, 2015	2015	Ann Pharmacother	Number (proportion) of patients for whom clinical pharmacists have completed (executed/implemented) a pharmaceutical care plan	NC	Process	
Fernandes, 2015	2015	Ann Pharmacother	Number of total drug therapy problems resolved by pharmacists	NC	Process	
Fernandes, 2015	2015	Ann Pharmacother	Number (or proportion) of patients receiving proactive comprehensive direct patient care by a pharmacist in collaboration with the health care team	NC	Process	
Fernandes, 2015	2015	Ann Pharmacother	Number (or proportion) of hospital patients who receive medication counseling by a pharmacist at discharge	NC	Process	
Fernandes, 2015	2015	Ann Pharmacother	Number (or proportion) of patients who have received in-person education from a pharmacist about their disease(s) and medication(s) during their hospital stay	NC	Process	
King, 2021	2021	JOURNAL OF THE AMERICAN COLLEGE OF CLINICAL PHARMACY	Proportion of patients within a total target population receiving specific TOC service(s) aimed at identifying medication discrepancies and medication therapy problems	Process	Process	

KPI classification				Original author classification	Review classification	
Author, year	Year published	Periodic	KPI Description	Type (Donabedian): • Structure (S) • Process (P) • Outcome (O) (NC = not classified)	Donabedian framework: • Structure (S) • Process (P) • Outcome (O)	Outcome classification (Kozma framework): - Economic (E) - Clinical (C) - Humanistic (H)
King, 2021	2021	JOURNAL OF THE AMERICAN COLLEGE OF CLINICAL PHARMACY	Medication discrepancies<br>◦ Total number and distribution, overall and by type	Process	Process	
King, 2021	2021	JOURNAL OF THE AMERICAN COLLEGE OF CLINICAL PHARMACY	Medication discrepancies and medication therapy problems<br>◦ Total number and distribution, overall and by type<br>◦ Percentage of patients with ≥1, overall and by type	Process	Process	
King, 2021	2021	JOURNAL OF THE AMERICAN COLLEGE OF CLINICAL PHARMACY	Resolution rate of medication discrepancies and medication therapy problems: total percent successfully resolved, overall and by type	Outcome	Process	
King, 2021	2021	JOURNAL OF THE AMERICAN COLLEGE OF CLINICAL PHARMACY	Completion rates of first planned follow-up contact within prespecified time intervals, including time until follow-up<br>◦ For example, completion of postdischarge telephone interview within 72 hours, completion of postdischarge ambulatory care visit within 7 days (primary care, specialty care, and/or other referrals)	Outcome	Process	
King, 2021	2021	JOURNAL OF THE AMERICAN COLLEGE OF CLINICAL PHARMACY	Completion rates of pharmacist-referred visits for preventive medicine or other medication-related concerns	Outcome	Process	

KPI classification				Original author classification	Review classification	
Author, year	Year published	Periodic	KPI Description	Type (Donabedian): • Structure (S) • Process (P) • Outcome (O) (NC = not classified)	Donabedian framework: • Structure (S) • Process (P) • Outcome (O)	Outcome classification (Kozma framework): - Economic (E) - Clinical (C) - Humanistic (H)
King, 2021	2021	JOURNAL OF THE AMERICAN COLLEGE OF CLINICAL PHARMACY	Clinician Satisfaction (including pharmacists)<br>◦ Satisfaction of clinicians with pharmacists and specific services	Outcome	Process	
King, 2021	2021	JOURNAL OF THE AMERICAN COLLEGE OF CLINICAL PHARMACY	Clinician engagement<br>◦ Readiness of clinicians to establish new services<br>◦ Rates of clinician engagement with new or existing services	Outcome	Process	
King, 2021	2021	JOURNAL OF THE AMERICAN COLLEGE OF CLINICAL PHARMACY	Patient engagement<br>◦ Medication adherence<br>▪ Verified prescription capture rates over time, such as through insurance claims data, including whether prescriptions were physically obtained, delivered, or administered to patients<br>▪ Verified medication adherence (taking >80% of prescribed doses for scheduled medications)<br>▪ Consider the percentage of days covered, percentage of patients taking >80% of prescribed doses, 8-item Morisky Medication Adherence Scale (MMAS-8), pill counts, or drug concentrations<br>◦ Patient Activation Assessment score<br>◦ Completion rates of prespecified follow-up appointments (eg, inperson, by telephone, or through telemedicine) with pharmacists or other providers	Outcome	Process	
King, 2021	2021	JOURNAL OF THE AMERICAN	Rates of unplanned 30-day, all-cause hospital readmissions	Outcome	Outcome	Clinical

KPI classification				Original author classification	Review classification	
Author, year	Year published	Periodic	KPI Description	Type (Donabedian): • Structure (S) • Process (P) • Outcome (O) (NC = not classified)	Donabedian framework: • Structure (S) • Process (P) • Outcome (O)	Outcome classification (Kozma framework): - Economic (E) - Clinical (C) - Humanistic (H)
		COLLEGE OF CLINICAL PHARMACY				
King, 2021	2021	JOURNAL OF THE AMERICAN COLLEGE OF CLINICAL PHARMACY	Rates of unplanned 30-day, all-cause hospital readmissions plus ED visits	Outcome	Outcome	Clinical
King, 2021	2021	JOURNAL OF THE AMERICAN COLLEGE OF CLINICAL PHARMACY	Total hospital length of stay (measured as hours or days) ◦ ICU and non-ICU portions should be included to assess TOC interventions during TOC within the hospital	Outcome	Outcome	Clinical
King, 2021	2021	JOURNAL OF THE AMERICAN COLLEGE OF CLINICAL PHARMACY	Rates of unplanned Health care utilization (HCU) within various time points, including medication-related causes	Outcome	Outcome	Clinical
King, 2021	2021	JOURNAL OF THE AMERICAN COLLEGE OF CLINICAL PHARMACY	Patient satisfaction ◦ HCAHPS or CAHPS scores (for Medicare and Medicaid populations) ▪ Specifically, with respect to survey items pertaining to medications ◦ Items adapted from CMS surveys or other validated surveys aimed at specific TOC services within specific TOC settings ◦ Press Ganey ◦ Care Transitions Measure	Outcome	Outcome	Humanistic

KPI classification				Original author classification	Review classification	
Author, year	Year published	Periodic	KPI Description	Type (Donabedian): • Structure (S) • Process (P) • Outcome (O) (NC = not classified)	Donabedian framework: • Structure (S) • Process (P) • Outcome (O)	Outcome classification (Kozma framework): - Economic (E) - Clinical (C) - Humanistic (H)
King, 2021	2021	JOURNAL OF THE AMERICAN COLLEGE OF CLINICAL PHARMACY	Return on investment (ROI): Net revenue generated OR the ratio of total cost savings to total cost of servicea<br>◦ Direct and indirect costs of providing the service<br>▪ Cost per unit of time spent by each discipline involved<br>▪ Cost of physical resources necessary to provide the service<br>▪ Cost of training, administrative responsibilities, and other overhead costs<br>◦ Direct and indirect cost savings<br>▪ Cost saved from interventions focused on minimizing institutional costs while maintaining or optimizing drug therapy<br>▪ Cost saved to institutions or payers, such as through interventions that help avoid financial penalties from excessive readmission rates, reduce hospital length of stay, or help maintain quality ratings	Outcome	Outcome	Economic
King, 2021	2021	JOURNAL OF THE AMERICAN COLLEGE OF CLINICAL PHARMACY	• Direct cost savings<br>◦ Difference between actual and expected cost for a service, medication, or other health care-related resource (eg, planned vs unplanned HCU, avoided health care costs after adverse drug reactions)	Outcome	Outcome	Economic
Krzyżaniak, 2018	2018	Int J Clin Pharm	Availability of written policies/protocols/guidelines for high-risk medications i.e. antibiotics, pain-relief, parenteral nutrition	Structure	Structure	
Krzyżaniak, 2018	2018	Int J Clin Pharm	Availability of clear policies on how to prescribe, dispense, administer and monitor medications in the NICU	Structure	Structure	

KPI classification				Original author classification	Review classification	
Author, year	Year published	Periodic	KPI Description	Type (Donabedian): • Structure (S) • Process (P) • Outcome (O) (NC = not classified)	Donabedian framework: • Structure (S) • Process (P) • Outcome (O)	Outcome classification (Kozma framework): - Economic (E) - Clinical (C) - Humanistic (H)
Krzyżaniak, 2018	2018	Int J Clin Pharm	Availability of emergency medicines sheets, with listed doses per weight	Structure	Structure	
Krzyżaniak, 2018	2018	Int J Clin Pharm	Availability of standard neonatal/pediatric references for use in the selection, use and evaluation of medications i.e. textbooks (BNF P, Neofax), online resources	Structure	Structure	
Ramos, 2023	2023	Emergencias	Availability of circuits and procedures for the pharmacokinetic monitoring of high-risk medications.	NC	Structure	
Ramos, 2023	2023	Emergencias	Report antimicrobial consumption in the emergency setting.	NC	Structure	
Krzyżaniak, 2018	2018	Int J Clin Pharm	Availability of electronic medication error and adverse drug event reporting (systems)	Structure	Structure	
Lopes, 2021	2021	Int J Clin Pharm	Percentage of services with pharmacist rounds	NC	Structure	
Lopes, 2021	2021	Int J Clin Pharm	Existence of specifc outpatient pharmaceutical consultations (Identify which specialties)	NC	Structure	
Krzyżaniak, 2018	2018	Int J Clin Pharm	Proportion of unlicensed/of-label prescriptions that involved the consultation of a pharmacist	Process	Process	
Krzyżaniak, 2018	2018	Int J Clin Pharm	Proportion of adverse drug events that were identifed, monitored, rectifed, prevented, and reported per number of admissions	Process	Process	
Krzyżaniak, 2018	2018	Int J Clin Pharm	Proportion of dispensing errors identifed and rectifed by pharmacist per number of admissions	Process	Process	
Krzyżaniak, 2018	2018	Int J Clin Pharm	Number of pharmacotherapy related consultations provided to medical personnel by pharmacists	Process	Process	
Krzyżaniak, 2018	2018	Int J Clin Pharm	Proportion of TPN regimens that have been monitored/optimized by a pharmacist	Process	Process	
Krzyżaniak, 2018	2018	Int J Clin Pharm	Proportion of IV medications that have been monitored by a pharmacist	Process	Process	

KPI classification				Original author classification	Review classification	
Author, year	Year published	Periodic	KPI Description	Type (Donabedian): • Structure (S) • Process (P) • Outcome (O) (NC = not classified)	Donabedian framework: • Structure (S) • Process (P) • Outcome (O)	Outcome classification (Kozma framework): - Economic (E) - Clinical (C) - Humanistic (H)
Krzyżaniak, 2018	2018	Int J Clin Pharm	Proportion of dose calculations checked by pharmacist before administration	Process	Process	
Krzyżaniak, 2018	2018	Int J Clin Pharm	Proportion of patients whose therapy is being monitored by a pharmacist	Process	Process	
Krzyżaniak, 2018	2018	Int J Clin Pharm	Proportion of extemporaneous medications that have been prepared and monitored by a pharmacist for the NICU	Process	Process	
Krzyżaniak, 2018	2018	Int J Clin Pharm	Percentage of medication orders that include the correct dose per kilogram (or body surface area) AND an effective and safe total dose	Outcome	Process	
Krzyżaniak, 2018	2018	Int J Clin Pharm	Medication error rates/reports per 6 months	Outcome	Process	
Krzyżaniak, 2018	2018	Int J Clin Pharm	Monthly audit of episodes of antibiotic-associated adverse events	Outcome	Outcome	Clinical
Krzyżaniak, 2018	2018	Int J Clin Pharm	Adverse drug event rates/reports per 6 months	Outcome	Outcome	Clinical
Krzyżaniak, 2018	2018	Int J Clin Pharm	Costs of therapy	Outcome	Outcome	Economic
Lopes, 2021	2021	Int J Clin Pharm	Existence of written information regarding prescribed medications at discharge (yes / no)	NC	Structure	
Lopes, 2021	2021	Int J Clin Pharm	Existence of written information regarding outpatients prescribed medications (yes / no)	NC	Structure	
Ramos, 2023	2023	Emergencias	Availability of a conciliation program in the emergency department.	NC	Structure	
Ramos, 2023	2023	Emergencias	High conciliation program available.	NC	Structure	
Lopes, 2021	2021	Int J Clin Pharm	Number of inpatients with therapeutic reconciliation, adjusted by pharmacist FTE	NC	Process	
Lopes, 2021	2021	Int J Clin Pharm	Number of inpatient prescriptions validations (medication review), adjusted by pharmacist FTE	NC	Process	
Lopes, 2021	2021	Int J Clin Pharm	Existence of medication reconciliations up to 72 h after admission (yes / no)	NC	Process	

KPI classification				Original author classification	Review classification	
Author, year	Year published	Periodic	KPI Description	Type (Donabedian): • Structure (S) • Process (P) • Outcome (O) (NC = not classified)	Donabedian framework: • Structure (S) • Process (P) • Outcome (O)	Outcome classification (Kozma framework): - Economic (E) - Clinical (C) - Humanistic (H)
Lopes, 2021	2021	Int J Clin Pharm	Existence of medication reconciliations at discharge (Yes / No)	NC	Process	
Lopes, 2021	2021	Int J Clin Pharm	Number of outpatient prescription validations (medication review), adjusted by pharmacist FTE	NC	Process	
Lopes, 2021	2021	Int J Clin Pharm	Number of pharmacist interventions in patient therapy, adjusted by pharmacist FTE	NC	Process	
Lopes, 2021	2021	Int J Clin Pharm	Number of blood products orders analysed, per 1000 patients discharged	NC	Process	
Lopes, 2021	2021	Int J Clin Pharm	Number of blood products orders dispensed, per 1000 patients discharged	NC	Process	
Lopes, 2021	2021	Int J Clin Pharm	Number of blood products orders analyzed, per 1000 patients discharged	NC	Process	
Lopes, 2021	2021	Int J Clin Pharm	Number of narcotic and psychotropic requests analysed, per 1000 patients discharged	NC	Process	
Lopes, 2021	2021	Int J Clin Pharm	Number of narcotic and psychotropic requests dispensed, per 1000 patients discharged	NC	Process	
Lopes, 2021	2021	Int J Clin Pharm	Number of outpatient pharmaceutical consultations, adjusted by pharmacist FTE	NC	Process	
Ng, 2010	2010	Journal of Pharmaceutical Health Services Research	KPI02 Medication reconciliation: Proportion of patients for whom medication reconciliation is undertaken and discrepancies identified are resolved	NC	Process	
Ng, 2010	2010	Journal of Pharmaceutical Health Services Research	KPI45 Proportion of patients with a toxic or sub-therapeutic aminoglycoside concentration whose dosage has been adjusted or reviewed prior to the next dose	NC	Process	

KPI classification				Original author classification	Review classification	
Author, year	Year published	Periodic	KPI Description	Type (Donabedian): • Structure (S) • Process (P) • Outcome (O) (NC = not classified)	Donabedian framework: • Structure (S) • Process (P) • Outcome (O)	Outcome classification (Kozma framework): - Economic (E) - Clinical (C) - Humanistic (H)
Ng, 2010	2010	Journal of Pharmaceutical Health Services Research	KPI27 Proportion of paediatric medication orders that include the correct dose per kilogram (or body surface area) AND a safe total dose	NC	Process	
Ng, 2010	2010	Journal of Pharmaceutical Health Services Research	KPI05 Proportion of patients discharged on warfarin that receive written information regarding warfarin management prior to discharge	NC	Process	
Ng, 2010	2010	Journal of Pharmaceutical Health Services Research	KPI21 Chart review: Proportion of medicine charts reviewed by clinical pharmacists within 24 hours of admission	NC	Process	
Ng, 2010	2010	Journal of Pharmaceutical Health Services Research	KPI42 Proportion of patients with an INR above 4 whose dosage has been adjusted or reviewed prior to the next warfarin dose	NC	Process	
Ng, 2010	2010	Journal of Pharmaceutical Health Services Research	KPI30 Proportion of patients receiving appropriate initial antibiotic selection for CAP (Community acquired pneumonia)	NC	Process	
Ng, 2010	2010	Journal of Pharmaceutical Health Services Research	KPI35 Proportion of patients undergoing specified surgical procedures that receive an appropriate prophylactic antibiotic regimen	NC	Process	
Ng, 2010	2010	Journal of Pharmaceutical Health Services Research	KPI28 Proportion of medication orders for intermittent therapy that are prescribed safely (e.g. alternate days or once weekly regimen)	NC	Process	

KPI classification				Original author classification	Review classification	
Author, year	Year published	Periodic	KPI Description	Type (Donabedian): • Structure (S) • Process (P) • Outcome (O) (NC = not classified)	Donabedian framework: • Structure (S) • Process (P) • Outcome (O)	Outcome classification (Kozma framework): - Economic (E) - Clinical (C) - Humanistic (H)
Ng, 2010	2010	Journal of Pharmaceutical Health Services Research	KPI34 Proportion of patients at high risk of venous thromboembolism that receive appropriate prophylaxis	NC	Process	
Ng, 2010			KPI01 Accurate medication history: Proportion of patients with an accurate documented record of their medication taking behaviour	NC	Process	
Ng, 2010			KPI15 Chemotherapy: Proportions of patients receiving cytotoxic chemotherapy whose treatment is guided by a hospital approved chemotherapy treatment protocol	NC	Process	
Ng, 2010			KPI16 Proportion of patients prescribed hospital initiated warfarin whose loading doses are consistent with a hospital approved protocol	NC	Process	
Ng, 2010			KPI17 Proportion of patients presenting with community acquired pneumonia that are prescribed guideline concordant antibiotic therapy	NC	Process	
Ng, 2010			KPI18 Proportion of prescriptions for restricted antibiotics that are concordant with hospital approved criteria	NC	Process	
Ng, 2010			KPI22 Proportion of patients that are reviewed by a clinical pharmacist within 24 hours of admission	NC	Process	
Ng, 2010			KPI23 Pharmaceutical care plan: Proportion of patients where clinical pharmacists have completed a documented comprehensive care plan on the medicines regimen	NC	Process	
Ng, 2010			KPI24 Clinical pharmacy interventions: Identification and resolution of potential or actual drug related problems per patient bed day	NC	Process	

KPI classification				Original author classification	Review classification	
Author, year	Year published	Periodic	KPI Description	Type (Donabedian): • Structure (S) • Process (P) • Outcome (O) (NC = not classified)	Donabedian framework: • Structure (S) • Process (P) • Outcome (O)	Outcome classification (Kozma framework): - Economic (E) - Clinical (C) - Humanistic (H)
Ng, 2010			KPI25 Prescribing errors: Identification and resolution of unintentional departure from recommended prescribing practices per patient bed day	NC	Process	
Ng, 2010			KPI37 Documented allergy status: Proportion of patients with incomplete allergy status at any point	NC	Process	
Ng, 2010			KPI38 Patient counselling: Proportion of patients who have had a face-to-face discussion about medicines-specific information	NC	Process	
Ng, 2010			KPI39 Medication card provision: Proportion of patients provided with a medicines information card containing the patient's current medicines	NC	Process	
Ng, 2010			KPI40 Discharge counselling: Proportion of patients that have had a formal discussion about their medicines immediately prior to discharge	NC	Process	
Ng, 2010			KPI41 Administration errors: Identification and resolution of unintended departure from recommended administration practices per patient bed day	NC	Process	
Ng, 2010			KPI43 Adverse drug reaction monitoring: Prevention, detection, assessment, management, appropriate reporting and documentation of adverse drug reactions per number of admissions	NC	Process	
Ramos, 2023	2023	Emergencias	Medication reconciliation program/protocol in the emergency department.	NC	Structure	
Ramos, 2023	2023	Emergencias	Participation in programs optimization programs for the use of antimicrobials in the Emergency Department	NC	Structure	
Krzyżaniak, 2018	2018	Int J Clin Pharm	Availability of suitable fridges for vaccines and TPN on the ward	Structure	Structure	

KPI classification				Original author classification	Review classification	
Author, year	Year published	Periodic	KPI Description	Type (Donabedian): • Structure (S) • Process (P) • Outcome (O) (NC = not classified)	Donabedian framework: • Structure (S) • Process (P) • Outcome (O)	Outcome classification (Kozma framework): - Economic (E) - Clinical (C) - Humanistic (H)
Krzyżaniak, 2018	2018	Int J Clin Pharm	Direct availability on the ward of essential medicines for specific use within the NICU	Structure	Structure	
Krzyżaniak, 2018	2018	Int J Clin Pharm	Availability of a funded NICU clinical pharmacist position (full-time/part-time) in the hospital	Structure	Structure	
Krzyżaniak, 2018	2018	Int J Clin Pharm	NICU pharmacist holds qualifications in clinical pharmacy or NICU/pediatric pharmacy	Structure	Structure	
Ramos, 2023	2023	Emergencias	Number of proposed interventions.	NC	Process	
Ramos, 2023	2023	Emergencias	% validation in electronic prescription program.	NC	Process	
Ramos, 2023	2023	Emergencias	% acceptance of proposed interventions.	NC	Process	
Ramos, 2023	2023	Emergencias	Number of consultations received.	NC	Process	
Ramos, 2023	2023	Emergencias	Number of medications reconciled.	NC	Process	
Ramos, 2023	2023	Emergencias	Number of modified discharge prescriptions.	NC	Process	
Ramos, 2023	2023	Emergencias	Number of modified discharge prescriptions.	NC	Process	
Ramos, 2023	2023	Emergencias	Validation percentage upon admission and discharge.	NC	Process	
Ramos, 2023	2023	Emergencias	% Number of justified discrepancies detected.	NC	Process	
Ramos, 2023	2023	Emergencias	Number of pharmacokinetic recommendations made.	NC	Process	
Ramos, 2023	2023	Emergencias	Participation in clinical rounds of patient evaluation.	NC	Process	
Ramos, 2023	2023	Emergencias	Participation in pain management adaptation programs.	NC	Process	
Ramos, 2023	2023	Emergencias	Participation in sepsis code codes/protocols.	NC	Process	
Ramos, 2023	2023	Emergencias	Number of interventions performed in sepsis code.	NC	Process	
Ramos, 2023	2023	Emergencias	Participation in cardiopulmonary resuscitation codes/protocols.	NC	Process	
Ramos, 2023	2023	Emergencias	Number of interventions performed in heart attack code.	NC	Process	
Ramos, 2023	2023	Emergencias	Participation in stroke code codes/protocols.	NC	Process	
Ramos, 2023	2023	Emergencias	Number of interventions performed in stroke code.	NC	Process	
Ramos, 2023	2023	Emergencias	Participation in polytrauma code codes/protocols.	NC	Process	
Ramos, 2023	2023	Emergencias	Number of interventions performed in polytrauma code.	NC	Process	

KPI classification				Original author classification	Review classification	
Author, year	Year published	Periodic	KPI Description	Type (Donabedian): • Structure (S) • Process (P) • Outcome (O) (NC = not classified)	Donabedian framework: • Structure (S) • Process (P) • Outcome (O)	Outcome classification (Kozma framework): - Economic (E) - Clinical (C) - Humanistic (H)
Anene-Okeke, 2022	2022	Journal of Basic and Clinical Pharmacy	Number of patients who receive formal documented admission medication reconciliation by a pharmacist	NC	Process	
Anene-Okeke, 2022	2022	Journal of Basic and Clinical Pharmacy	Number of pharmacists who actively participate in inter professional patient care rounds to improve medication management	NC	Process	
Anene-Okeke, 2022	2022	Journal of Basic and Clinical Pharmacy	Number of patients for whom clinical pharmacists have completed (executed/implemented) a pharmaceutical care plan	NC	Process	
Anene-Okeke, 2022	2022	Journal of Basic and Clinical Pharmacy	Number of total drug therapy problems resolved by pharmacists	NC	Process	
Anene-Okeke, 2022	2022	Journal of Basic and Clinical Pharmacy	Number of patients receiving comprehensive direct patient care by a pharmacist in collaboration with the health care team.	NC	Process	
Anene-Okeke, 2022	2022	Journal of Basic and Clinical Pharmacy	Proportion of patients who receive formal documented discharge medication reconciliation and resolution of identified discrepancies by a pharmacist	NC	Process	
Anene-Okeke, 2022	2022	Journal of Basic and Clinical Pharmacy	Number of hospital patients who receive medication counselling by a pharmacist at discharge	NC	Process	
Anene-Okeke, 2022	2022	Journal of Basic and Clinical Pharmacy	Number of patients who have received education from a pharmacist about their disease(s) and medication(s) during their hospital stay	NC	Process	
Anene-Okeke, 2022	2022	Journal of Basic and Clinical Pharmacy	Proportion of medicine charts reviewed by clinical pharmacists within 24 hours of admission	NC	Process	

KPI classification				Original author classification	Review classification	
Author, year	Year published	Periodic	KPI Description	Type (Donabedian): • Structure (S) • Process (P) • Outcome (O) (NC = not classified)	Donabedian framework: • Structure (S) • Process (P) • Outcome (O)	Outcome classification (Kozma framework): - Economic (E) - Clinical (C) - Humanistic (H)
Anene-Okeke, 2022	2022	Journal of Basic and Clinical Pharmacy	Number of patients who have a complete and accurate list of their current medications (including over the counter and complementary medications) documented	NC	Process	
Anene-Okeke, 2022	2022	Journal of Basic and Clinical Pharmacy	The number of drug information enquiries that have been answered	NC	Process	
Anene-Okeke, 2022	2022	Journal of Basic and Clinical Pharmacy	Number of attempted clinical interventions by the pharmacists that were accepted by the clinician	NC	Process	
Anene-Okeke, 2022	2022	Journal of Basic and Clinical Pharmacy	The number of ward meetings attended by the pharmacist	NC	Process	
Anene-Okeke, 2022	2022	Journal of Basic and Clinical Pharmacy	Total number of patients reviewed by clinical pharmacy services per month	NC	Process	
Anene-Okeke, 2022	2022	Journal of Basic and Clinical Pharmacy	Number of medication errors reported	NC	Process	
Anene-Okeke, 2022	2022	Journal of Basic and Clinical Pharmacy	Number of instances of in-service education (Journal clubs, Staff education)	NC	Process	
Anene-Okeke, 2022	2022	Journal of Basic and Clinical Pharmacy	Number of students/ Residents perecepted	NC	Process	
Anene-Okeke, 2022	2022	Journal of Basic and Clinical Pharmacy	Number of reviews for guidelines and protocols	NC	Process	

KPI classification				Original author classification	Review classification	
Author, year	Year published	Periodic	KPI Description	Type (Donabedian): • Structure (S) • Process (P) • Outcome (O) (NC = not classified)	Donabedian framework: • Structure (S) • Process (P) • Outcome (O)	Outcome classification (Kozma framework): - Economic (E) - Clinical (C) - Humanistic (H)
Anene-Okeke, 2022	2022	Journal of Basic and Clinical Pharmacy	Number of patients who have a correctly completed record (medication and reaction) of prior Adverse Drug Reaction (ADR) and allergy documented	NC	Process	
Anene-Okeke, 2022	2022	Journal of Basic and Clinical Pharmacy	Prescribing errors: Identification and resolution of unintentional departure from recommended prescribing practices	NC	Process	
Anene-Okeke, 2022	2022	Journal of Basic and Clinical Pharmacy	The number of complaints that pharmacy department has received	NC	Outcome	Humanistic
Aljamal, 2016	2016	International Journal of Pharmacy Practice	Number of patients whose drug history was compared for accuracy (correctness and completeness) with drugs prescribed on admission as a percentage of the number of patients reconciled over a defined period of time.	NC	Process	
Aljamal, 2016	2016	International Journal of Pharmacy Practice	Number of patients whose drug allergies were checked as percentage of the number of patients reconciled over a defined period of time.	NC	Process	
Aljamal, 2016	2016	International Journal of Pharmacy Practice	Number of patients for whom the names of all drugs to which they were allergic was documented as percentage of the number of patients reconciled with identified allergies over a defined period of time.	NC	Process	
Aljamal, 2016	2016	International Journal of Pharmacy Practice	Number of patients admitted to hospital whose medicines were reconciled within 24 h as a percentage of the number of patients admitted over a defined period of time.	NC	Process	
Aljamal, 2016	2016	International Journal of	Number of patients whose medication history was taken on admission (by pharmacy staff) as a percentage of the	NC	Process	

KPI classification				Original author classification	Review classification	
Author, year	Year published	Periodic	KPI Description	Type (Donabedian): • Structure (S) • Process (P) • Outcome (O) (NC = not classified)	Donabedian framework: • Structure (S) • Process (P) • Outcome (O)	Outcome classification (Kozma framework): - Economic (E) - Clinical (C) - Humanistic (H)
		Pharmacy Practice	number of patients admitted over a defined period of time.			
Aljamal, 2016	2016	International Journal of Pharmacy Practice	Number of patients whose medication history was completed within 24 h (by pharmacy staff) as a percentage of the number of patients admitted over a defined period of time.	NC	Process	
Aljamal, 2016	2016	International Journal of Pharmacy Practice	Number of patients' drug histories that were checked using more than one source (including patient and/or GP sources) as a percentage of the number of patients reconciled over a defined period of time.	NC	Process	
Aljamal, 2016	2016	International Journal of Pharmacy Practice	Number of patients for whom a drug allergy reaction was documented as a percentage of the number of patients with identified allergies reconciled over a defined period of time.	NC	Process	
Aljamal, 2016	2016	International Journal of Pharmacy Practice	Number of patients for whom medication discrepancies were identified as a percentage of the number of patients reconciled over a defined period of time.	NC	Process	
Aljamal, 2016	2016	International Journal of Pharmacy Practice	Number of patients whose unintentional discrepancies were identified (involving strength, dose and frequency) as a percentage of the number of patients reconciled over a defined period of time.	NC	Process	
Aljamal, 2016	2016	International Journal of Pharmacy Practice	Number of total discrepancies identified per 100 admissions reconciled.	NC	Process	
Aljamal, 2016	2016	International Journal of	Number of unintentional discrepancies identified per 100 admissions reconciled.	NC	Process	

KPI classification				Original author classification	Review classification	
Author, year	Year published	Periodic	KPI Description	Type (Donabedian): • Structure (S) • Process (P) • Outcome (O) (NC = not classified)	Donabedian framework: • Structure (S) • Process (P) • Outcome (O)	Outcome classification (Kozma framework): - Economic (E) - Clinical (C) - Humanistic (H)
		Pharmacy Practice				
Aljamal, 2016	2016	International Journal of Pharmacy Practice	Number of unintentional discrepancies that reached patients (identified by checking the nurse's signature on the drug chart) as a percentage of the number of unintentional discrepancies over a defined period of time.	NC	Process	
Aljamal, 2016	2016	International Journal of Pharmacy Practice	Number of patients whose medication reconciliation process was documented (using any form of documentation) as a percentage of the number of patients reconciled over a defined period of time.	NC	Process	
Aljamal, 2016	2016	International Journal of Pharmacy Practice	Number of patients whose completed medication reconciliation information was available to staff taking care of them (doctors, nurses and pharmacy staff) as a percentage of the number of patients reconciled over a defined period of time.	NC	Process	
Aljamal, 2016	2016	International Journal of Pharmacy Practice	Number of patients admitted to hospital whose medicines are reconciled within 48 h as a percentage of the number of patients admitted over a defined period of time.	NC	Process	
Aljamal, 2016	2016	International Journal of Pharmacy Practice	Number of patients admitted to hospital whose medicines are reconciled within 72 h as a percentage of the number of patients admitted over a defined period of time.	NC	Process	
Aljamal, 2016	2016	International Journal of Pharmacy Practice	Number of patients for whom the medication reconciliation process was not performed as a percentage of the number of patients admitted over a defined period of time.	NC	Process	

KPI classification				Original author classification	Review classification	
Author, year	Year published	Periodic	KPI Description	Type (Donabedian): • Structure (S) • Process (P) • Outcome (O) (NC = not classified)	Donabedian framework: • Structure (S) • Process (P) • Outcome (O)	Outcome classification (Kozma framework): - Economic (E) - Clinical (C) - Humanistic (H)
Aljamal, 2016	2016	International Journal of Pharmacy Practice	Number of patients for whom the reasons for not performing medication reconciliation were identified (documented) as a percentage of the number of patients who had not received medication reconciliation at admission over a defined period of time.	NC	Process	
Aljamal, 2016	2016	International Journal of Pharmacy Practice	The time (in minutes) taken for completing the medication reconciliation process for patients reconciled over a defined period of time (total time required for medication reconciliation divided by number of patients reconciled).	NC	Process	
Aljamal, 2016	2016	International Journal of Pharmacy Practice	Number of patients whose drug history was performed by contacting the GP (doctor or receptionist) via phone to receive a drug history as a percentage of the number of patients reconciled over a defined period of time.	NC	Process	
Aljamal, 2016	2016	International Journal of Pharmacy Practice	Number of patients whose drug history was performed using a recent GP letter and/or repeat prescription for current admission (within one month) as a percentage of the number of patients reconciled over a defined period of time.	NC	Process	
Aljamal, 2016	2016	International Journal of Pharmacy Practice	Number of patients who were interviewed to verify their drug history as a percentage of the number of patients reconciled over a defined period of time.	NC	Process	
Aljamal, 2016	2016	International Journal of Pharmacy Practice	Number of patients whose drug history was performed using patient's own drugs (PODs) brought into hospital as a percentage of the number of patients who brought their own medication on admission over a defined period of time.	NC	Process	

KPI classification				Original author classification	Review classification	
Author, year	Year published	Periodic	KPI Description	Type (Donabedian): • Structure (S) • Process (P) • Outcome (O) (NC = not classified)	Donabedian framework: • Structure (S) • Process (P) • Outcome (O)	Outcome classification (Kozma framework): - Economic (E) - Clinical (C) - Humanistic (H)
Aljamal, 2016	2016	International Journal of Pharmacy Practice	Number of patients whose drug histories involved checking their history of OTC medication use (pharmacy item or general sales list) as a percentage of the number of patients reconciled over a defined period of time.	NC	Process	
Aljamal, 2016	2016	International Journal of Pharmacy Practice	Number of patients whose drug histories involved checking for history of the use of complementary (herbal) medicines as a percentage of the number of patients reconciled over a defined period of time.	NC	Process	
Aljamal, 2016	2016	International Journal of Pharmacy Practice	Number of patients whose medication adherence was checked (via the patient, carer, relative or compliance aid, e.g. Venalink) as a percentage of the number of patients reconciled over a defined period of time.	NC	Process	
Aljamal, 2016	2016	International Journal of Pharmacy Practice	Number of patients for whom information about their medication adherence was communicated to the prescriber as a percentage of the number of patients who had adherence issues over a defined period of time.	NC	Process	
Aljamal, 2016	2016	International Journal of Pharmacy Practice	Number of drug history lists that included drug intolerance as a percentage of the number of drug history lists for patients reconciled over a defined period of time.	NC	Process	
Aljamal, 2016	2016	International Journal of Pharmacy Practice	Number of unintentional discrepancies involving medication omitted from the admission prescription as a percentage of the number of unintentional discrepancies identified as part of the medication reconciliation process over a defined period of time.	NC	Process	
Aljamal, 2016	2016	International Journal of	Number of unintentional discrepancies involving the addition of a medication to the admission prescription as a percentage of the number of unintentional	NC	Process	

KPI classification				Original author classification	Review classification	
Author, year	Year published	Periodic	KPI Description	Type (Donabedian): • Structure (S) • Process (P) • Outcome (O) (NC = not classified)	Donabedian framework: • Structure (S) • Process (P) • Outcome (O)	Outcome classification (Kozma framework): - Economic (E) - Clinical (C) - Humanistic (H)
		Pharmacy Practice	discrepancies identified as part of the medication reconciliation process over a defined period of time.			
Aljamal, 2016	2016	International Journal of Pharmacy Practice	Number of unintentional discrepancies involving a dose change in the admission prescription as a percentage of the number of unintentional discrepancies identified as part of the medication reconciliation process over a defined period of time.	NC	Process	
Aljamal, 2016	2016	International Journal of Pharmacy Practice	Number of times that unintentional discrepancies were changed (by pharmacy staff if authorised) in the medication chart as a percentage of the number of unintentional discrepancies over a defined period of time.	NC	Process	
Aljamal, 2016	2016	International Journal of Pharmacy Practice	Number of times that unintentional discrepancies were documented in the patient's medical record as a percentage of the number of unintentional discrepancies identified over a defined period of time.	NC	Process	
Aljamal, 2016	2016	International Journal of Pharmacy Practice	Number of patients whose drug history was performed by contacting the GP using fax to receive a drug history (or GP's electronic record) as a percentage of the number of patients reconciled over a defined period of time.	NC	Process	
Aljamal, 2016	2016	International Journal of Pharmacy Practice	Number of patients' carers or family members interviewed to check (clarify) drug history as a percentage of the number of patients who could not be communicated with and who had a contactable carer or family member over a defined period of time.	NC	Process	
Aljamal, 2016	2016	International Journal of	Number of patients whose drug history was performed using any of the updated hospital sources (such as	NC	Process	

KPI classification				Original author classification	Review classification	
Author, year	Year published	Periodic	KPI Description	Type (Donabedian): • Structure (S) • Process (P) • Outcome (O) (NC = not classified)	Donabedian framework: • Structure (S) • Process (P) • Outcome (O)	Outcome classification (Kozma framework): - Economic (E) - Clinical (C) - Humanistic (H)
		Pharmacy Practice	clinical notes of previous admissions, discharges, allergy data, or previous letters with documenting the date of the last update) as a percentage of the number of patients reconciled over a defined period of time.			
Aljamal, 2016	2016	International Journal of Pharmacy Practice	Number of patients whose drug history was performed using community pharmacy patient medication records (such as the Venalink compliance aid system) as a percentage of the number of patients reconciled over a defined period of time.	NC	Process	
Aljamal, 2016	2016	International Journal of Pharmacy Practice	Number of patients' drug history lists (obtained by pharmacy staff) that contained all information on medicines (drug name, strength, dose, frequency and route of administration) as a percentage of the number of drug history lists for patients reconciled over a defined period of time.	NC	Process	
Aljamal, 2016	2016	International Journal of Pharmacy Practice	Number of times that the prescriber was contacted to clarify discrepancies as a percentage of the number of patients with unintentional discrepancies identified over a defined period of time.	NC	Process	
Aljamal, 2016	2016	International Journal of Pharmacy Practice	Number of patients whose drug histories were compared with the admission prescriptions (by pharmacist) to check discrepancies as a percentage of the number of patients reconciled over a defined period of time.	NC	Process	
Lloyd, 2016	2016	Journal of Pharmacy Practice and Research	percentage of inpatients that have a correctly completed record (medication and reaction) of prior ADR and allergy documented within a day of admission	NC	Process	

KPI classification				Original author classification	Review classification	
Author, year	Year published	Periodic	KPI Description	Type (Donabedian): • Structure (S) • Process (P) • Outcome (O) (NC = not classified)	Donabedian framework: • Structure (S) • Process (P) • Outcome (O)	Outcome classification (Kozma framework): - Economic (E) - Clinical (C) - Humanistic (H)
Lloyd, 2016	2016	Journal of Pharmacy Practice and Research	percentage of hospital inpatients who receive verbal counselling and/or written information about their medicines prior to discharge	NC	Process	
Lloyd, 2016	2016	Journal of Pharmacy Practice and Research	percentage of discharge prescriptions reviewed and reconciled by a pharmacist prior to dispensing	NC	Process	
Lloyd, 2016	2016	Journal of Pharmacy Practice and Research	percentage of patients who have a complete and accurate list of their current medications (including over the counter and complementary medications) documented and verified within a day of admission	NC	Process	
Lloyd, 2016	2016	Journal of Pharmacy Practice and Research	percentage of discharge summaries that document an accurate medication list and the reasons for all medication therapy changes from medicines taken prior to admission	NC	Process	
Lloyd, 2016	2016	Journal of Pharmacy Practice and Research	number of clinical interventions (any action by a pharmacist that directly results in a change in patient management or therapy) performed per patient bed day	NC	Process	
Lloyd, 2016	2016	Journal of Pharmacy Practice and Research	proportion of medicine charts reviewed by clinical pharmacists within 24 h of admission.	NC	Process	
Shawahna, 2020	2020	Evidence-Based Complementary and Alternative Medicine	Number of medication and/or CAM (complementary and alternative medicine) related problems identified and addressed/resolved by pharmacists including contraindications, inappropriate doses (over- and/or	NC	Process	

KPI classification				Original author classification	Review classification	
Author, year	Year published	Periodic	KPI Description	Type (Donabedian): • Structure (S) • Process (P) • Outcome (O) (NC = not classified)	Donabedian framework: • Structure (S) • Process (P) • Outcome (O)	Outcome classification (Kozma framework): - Economic (E) - Clinical (C) - Humanistic (H)
			underdoses), allergies, interactions, duplications, omissions, vague/ambiguous orders, inappropriate routes of administration, inappropriate duration of therapy, and reported ineffective therapies.			
Shawahna, 2020	2020	Evidence-Based Complementary and Alternative Medicine	Number of patients who received documented medication and/or CAM reconciliation by pharmacists including the best possible medication/CAM history/review and/or had their medication and/or CAM-related problems and discrepancies identified and addressed/resolved.	NC	Process	
Shawahna, 2020	2020	Evidence-Based Complementary and Alternative Medicine	Number of patients who received direct, comprehensive, and/or collaborative care by pharmacists.	NC	Process	
Shawahna, 2020	2020	Evidence-Based Complementary and Alternative Medicine	Number of patients for whom pharmacists were involved in planning/preparing/implementing/executing/completing a therapeutic plan.	NC	Process	
Shawahna, 2020	2020	Evidence-Based Complementary and Alternative Medicine	Number of patients who received formal counseling/education on their diseases and/or medications/CAM by pharmacists at the time of admission, stay, transition of care, and/or discharge from the healthcare facility.	NC	Process	
Shawahna, 2020	2020	Evidence-Based Complementary and Alternative Medicine	Number of written complaints on the services delivered by pharmacists received per a predefined period of time.	NC	Process	

KPI classification				Original author classification	Review classification	
Author, year	Year published	Periodic	KPI Description	Type (Donabedian): • Structure (S) • Process (P) • Outcome (O) (NC = not classified)	Donabedian framework: • Structure (S) • Process (P) • Outcome (O)	Outcome classification (Kozma framework): - Economic (E) - Clinical (C) - Humanistic (H)
Shawahna, 2020	2020	Evidence-Based Complementary and Alternative Medicine	Number of errors committed by pharmacists per a predefined period of time.	NC	Process	
Shawahna, 2020	2020	Evidence-Based Complementary and Alternative Medicine	Number of multi-healthcare provider discussions/deliberations for the purpose of improving care of patients in which pharmacists actively participated and contributed including answering formal inquiries by other healthcare providers.	NC	Process	
